# B Cell Fcγ Receptor IIb Modulates Atherosclerosis in Male and Female Mice by Controlling Adaptive Germinal Center and Innate B-1-Cell Responses

**DOI:** 10.1161/ATVBAHA.118.312272

**Published:** 2019-05-16

**Authors:** Jayashree Bagchi-Chakraborty, Anna Francis, Toni Bray, Leanne Masters, Dimitrios Tsiantoulas, Meritxell Nus, James Harrison, Michelle Broekhuizen, Jennifer Leggat, Menna R. Clatworthy, Marion Espéli, Kenneth G.C. Smith, Christoph J. Binder, Ziad Mallat, Andrew P. Sage

**Affiliations:** 1 From the Division of Cardiovascular Medicine (J.B.-C., A.F., T.B., L.M., D.T., M.N., J.H., M.B., J.L., Z.M., A.P.S.), Department of Medicine, University of Cambridge, United Kingdom; 2 Division of Immunology (M.R.C., K.G.C.S., Z.M.), Department of Medicine, University of Cambridge, United Kingdom; 3 INSERM U1160, Institut de Recherche Saint-Louis, Saint Louis Hospital, Paris, France (M.E.); 4 Department of Laboratory Medicine, Medical University of Vienna, Austria (C.J.B.); 5 CeMM Research Center for Molecular Medicine of the Austrian Academy of Sciences, Vienna (C.J.B.); 6 Institut National de la Santé et de la Recherche Médicale, Universite Paris-Descartes, Paris Cardiovascular Research Center, and Université Paris-Descartes, France (Z.M.).

**Keywords:** atherosclerosis, cardiovascular diseases, germinal center, inflammation, plasma cells

## Abstract

**Objective—:**

Investigate the impact of modulating B cell FcγRIIb (Fcγ receptor IIb) expression on atherosclerosis.

**Approach and Results—:**

Western diet–induced atherosclerosis was assessed in *Ldlr*^−^^/−^ or *Apoe*^−/−^ mice with B cell–specific overexpression of FcγRIIb or with an FcγRIIb promoter mutation that alters FcγRIIb expression in germinal center (GC) B cells. In males, overexpression of FcγRIIb on B cells severely reduced activated, class switched B cell responses, as indicated by reductions in GC B cells, plasma cells, and serum IgG but not IgM antibodies. Male mice overexpressing FcγRIIb developed less atherosclerosis, suggesting a pathogenic role for GC B cell IgG responses. In support of this hypothesis, male mice with a promoter polymorphism-driven reduction in FcγRIIb on GC B cells but not plasma cells have a converse phenotype of enhanced GC responses and IgG2c antibodies and enhanced atherosclerosis. IgG2c significantly enhanced TNF (tumor necrosis factor) secretion by CD11b^+^ CD11c^+^ cells expressing the high-affinity receptor FcγRIV. In females, overexpression of FcγRIIb on B cells not only reduced GC B cell responses but also substantially reduced B-1 cells and IgM antibodies, which translated into acceleration of atherosclerosis. Promoter-driven reduction in FcγRIIb did not alter GC B cell responses in females and, therefore, had no impact on atherosclerosis.

**Conclusions—:**

B cell FcγRIIb differentially alters proatherogenic adaptive GC B cell and atheroprotective innate B-1 responses in male and female mice fed a western diet. Our results highlight the importance of a better understanding and ability to selectively target B cell responses in future immunotherapeutic approaches against human cardiovascular disease.

## Abstract 2

Supplemental Digital Content is available in the text.

**Highlights**

- The inhibitory IgG receptor FcγRIIb (Fcγ receptor IIb) controls B cell activation and plasma cell survival.
- In atherosclerotic mice, specific modulation of B cell FcγRIIb expression levels reveals FcγRIIb suppresses a pathogenic (and proatherogenic) germinal center B cell response in male mice.
- Germinal center B cells produce IgG2c antibodies that promote TNF (tumor necrosis factor) production by monocyte-derived macrophages/dendritic cells via FcγRIV, equivalent to IgG1-FcγRIIIA in humans.
- FcγRIIb also controls atheroprotective B1-cell responses; in female atherosclerotic mice, this function is more dominant and FcγRIIb overexpression resulted in enhanced atherosclerosis.

Despite significant progress in reducing mortality rates, the incidence and morbidity associated with cardiovascular disease, most commonly caused by underlying atherosclerosis, continues to represent a leading global health concern. Atherosclerosis is driven by an aberrant response to LDL (low-density lipoprotein) accumulation by both vascular wall cells and invading immune cells. The modification of LDL is hypothesized to drive these nonresolving inflammatory cycles. B cell responses have long been associated with atherosclerosis, particularly antibody responses to oxidized LDL.^1,2^ IgM antibodies to proinflammatory oxidation-specific epitopes are produced by innate-like B-1 cells, which exhibit atheroprotective properties.^3^ In contrast, autoantibody-driven autoimmune diseases, such as SLE (systemic lupus erythematosus), are associated with accelerated atherosclerosis progression, although the mechanisms linking the 2 diseases are still under investigation.^4^ Autoimmunity is associated with a break-in tolerance, that is, a failure of checkpoints during B cell or T cell activation and production of class-switched IgG, IgE, or IgA antibodies to autoantigens.^5^ B cell depletion therapy using anti-CD20 antibodies is effective against some autoimmune diseases and also mitigates cardiovascular disease in experimental models of atherosclerosis,^6,7^ myocardial infarction,^8^ abdominal aortic aneurysm,^9^ and hypertension.^10^

**See accompanying editorial on page 1269**

A major effector mechanism of IgG antibodies is binding FcγRs (Fcγ receptors) on the cell surface of immune cells, distinguishing IgG functions from other isotypes such as IgM. There are several activating receptors (FcγRI, III, and IV in mice) expressed on monocytes, macrophages, dendritic cells, and neutrophils, which promote proinflammatory functions via immunoreceptor tyrosine-based activation motif-mediated signaling pathways.^11^ In contrast, there is a single inhibitory receptor, FcγRIIb, which antagonizes activating FcγR signaling through its intracellular immunoreceptor tyrosine-based inhibitory motif domains.^12^ FcγRIIb is also expressed on B cells, where it regulates B cell responses at multiple points.^12^ In fact, in humans, the major subtype expressing FcγRIIb is B cells.^11^ Co-ligation of the B cell receptor and FcγRIIb by antigen-IgG immune complexes raises the activation threshold compared with soluble antigen, limiting B cell responses. On plasma cells and B-1 cells, FcγRIIb has a direct proapoptotic influence that is continuously counteracted by survival factors such as BAFF (B cell–activating factor) of the TNF (tumor necrosis factor) family.^13,14^ FcγRIIb genetic mutations are associated with SLE and also with total IgG levels in humans, suggesting an important role for FcγRIIb in B cell–dependent regulation of disease.^15,16^ Investigations into the role of FcγRIIb in atherosclerosis have to date focused on *Fcgr2b*^−/−^ mice, with conflicting reports. In contrast to several previous studies that reported enhanced atherosclerosis,^17,18^ Harmon et al^19^ found decreased atherosclerosis in *Fcgr2b*^−/−^ *Apoe*^−/−^ mice. A subsequent study from Merched et al^20^ using *Fcgr2b*^−/−^ bone marrow (BM) transfer into *Ldlr*^−/−^ mice found increased atherosclerosis, supporting the previous studies. Considering that FcγRIIb expression on different cell types could have divergent functions, alternative approaches are necessary to provide additional insights. Since opposing roles for different B cell subsets in atherosclerosis have been revealed in several recent studies,^21–25^ we investigated the role of B cell FcγRIIb in atherosclerotic plaque development. Our data implicate FcγRIIb on B cells as an important regulator of atherosclerosis and reveal novel insights into how B cells respond to and regulate atherosclerosis.

## Methods

The data that support the findings of this study are available from the corresponding author on reasonable request.

### Mice

*Ldlr*^−/−^ (002207) and *Apoe*^−/−^ (002052) mice were originally obtained from the Jackson laboratory. FcγRIIb-B cell transgenic (B^tg^) mice and FcγRIIb^ΔAP-1^ mice were previously described^13,26^; both strains (created using C57BL6 embryos) were crossed to *Apoe*^−/−^ mice. B^tg^ mice were maintained via transgenic×nontransgenic breeding. Control (FcγRIIb-WT; wild-type [WT]) mice were nontransgenic *Apoe*^−/−^ littermates. Heterozygous FcγRIIb-ΔAP-1 (+/ΔAP-1) mice were used as breeders, and data from control (BL6; +/+ or +/ΔAP-1) and homozygous mutant (ΔAP-1/ΔAP-1) littermates are presented. BM transfer experiments were performed as previously described.^27^ Mice received western diet (21% fat and 0.15% cholesterol; Scientific Diet Services) for 6 or 12 weeks. Mice from different experimental groups were co-housed for atherosclerosis experiments. For IgM half-life experiments, male and female *Apoe*^−/−^ mice were injected intravenously with 200 μL of sex-matched Balb/c mouse serum (with normalized IgM concentration). Blood samples were collected 4, 25, and 48 hours post-injection from the saphenous vein. Mice were kept under a 12/12 hour light/dark cycle with access to food (DS150; SAFE) and water ad libitum. Cage bedding was from Datesand. All experiments were conducted under license and within the institutional guidelines of the University of Cambridge and adhered to the recommendations set out by the American Heart Association.

### Plaque and Serum Analysis

To assess plaque size and composition, aortic root cryosections were stained with Oil red O, hematoxylin and eosin, picrosirius red (All Sigma) or with anti-α–smooth muscle actin (Sigma), anti-CD3 (Dako) or MOMA2 (Biorad) antibodies. Images were analyzed using ImageJ (National Institutes of Health). Cellularity was determined by quantifying the proportion of nuclear-stained area within plaques (Hoechst staining). Serum total and oxidation-specific epitope antibodies were analyzed using kits from Bethyl labs or as previously described.^28^ Total cholesterol was quantified using a cholesterol RTU kit (Biomerieux) and serum BAFF by ELISA (BioTechne).

### Immune Cell Phenotyping

Flow cytometry of single cell suspensions was performed as previously described. Flow cytometry antibodies are detailed in Table I in the online-only Data Supplement, and gating strategies are outlined in Figure I in the online-only Data Supplement. In some experiments, B-1 cells were alternatively gated as B220^lo^ IgM^+^ CD43^+^ CD5^+^ and germinal center (GC) B cells as B220^+^ IgM^+^ CD95^+^ GL-7^+^. In vitro, B cell proliferation was assayed using splenic B cells purified by negative selection (Miltenyi) after stimulation for 72 hours with anti-IgM whole IgG or Fab’ fragments (Southern Biotech). B-1 cells were enriched by fluorescence-activated cell sorting from peritoneum as B220^+^ IgM^+^ CD43^+^ CD23^−^ cells. Apoptosis was assessed using an annexin V kit (Life technologies) and flow cytometry. Polystyrene beads (BD) were spiked into cultures before staining and used to normalize samples so that absolute numbers of remaining live cells (Annexin V^−^ propidium iodide^−^) could be quantified. IgM production was stimulated in B-1 cell cultures by incubating with IL-5 (5 ng/mL; Peprotech) and BAFF (10 ng/mL; Biotechne). CD11c^+^ CD11b^+^ macrophages/dendritic cells were generated with GM-CSF (granulocyte-monocyte colony stimulating factor; 20 ng/mL; BioLegend) and stimulated with IFNγ (interferon-γ; 2 ng/mL; BioLegend) in 96-well plates coated with mouse IgG2c or IgG1 (BioLegend) or treated with polystyrene beads (anti-mouse κ compbeads; BD) coated with mouse IgG2c or IgG1. Supernatants were analyzed for TNF levels by ELISA (Biotechne) after 48 hours.

### Statistical Analysis

Data were analyzed in GraphPad Prism (La Jolla, CA); technical dropout during data collection is indicated in the figure legends. Statistically significant outliers (Grubb test) were excluded where indicated in the figure legends. Two group comparisons were analyzed using unpaired *t* test or Mann-Whitney *U* test for data sets not passing normality (D’Agostino and Pearson test) or not having equal variances (*F* Test). Experiments with 3 or more groups were analyzed using 1-way ANOVA with Newman-Keuls multiple comparison test. Plaque size profiles were analyzed using 2-way ANOVA. A *P*<0.05 was considered significant. The number of asterisks indicate the *P* level (*<0.05, **<0.01, ***<0.005, ****<0.0001).

## Results

### B Cell Overexpression of FcγRIIb in Male Mice Reduces Atherosclerosis

**Figure 1.**
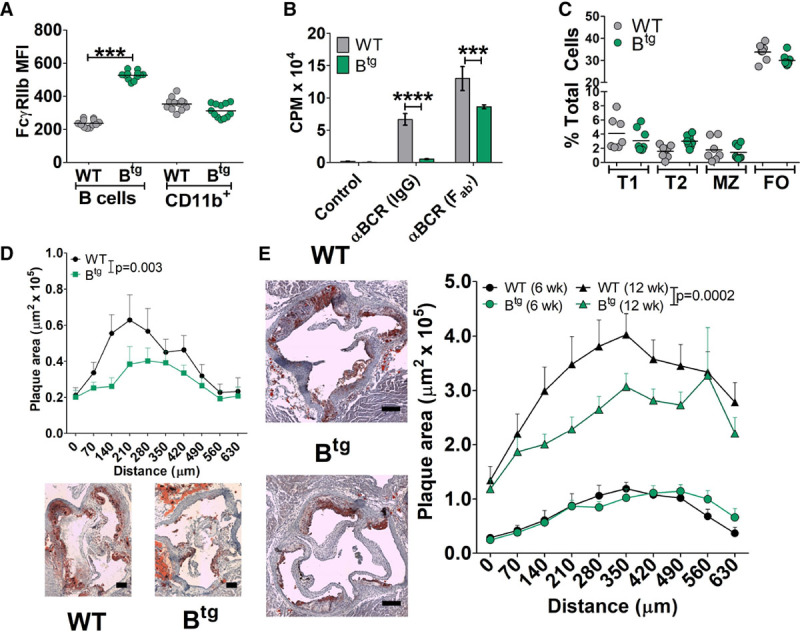
B cell overexpression of FcγRIIb (Fcγ receptor IIb) inhibits B cell activation and reduces atherosclerosis in males. **A**, Mean fluorescence intensity (MFI) for anti-FcγRIIb staining on B cells (B220^+^ IgM^+^) or CD11b^+^ cells from *Apoe*^−^^/−^ or B^tg^ *Apoe*^−/−^ mice. **B**, Proliferation of purified B cells from *Apoe*^−/−^ or B^tg^ *Apoe*^−/−^ mice after 72 h stimulation with anti-IgM whole IgG or Fab’ fragments. **C**, Levels of spleen B cell subsets (see Methods and Figure I in the online-only Data Supplement) in Ap*oe*^−/−^ or B^tg^ *Apoe*^−/−^ mice. **D** and **E**, Atherosclerosis quantified using Oil Red O–stained aortic root cryosections from male Ldlr^−/−^ mice transplanted with WT or B^tg^ bone marrow and fed western diet for 6 wk (**D**; n=12/group) or Apoe^−/−^ or B^tg^ Apoe^−/−^ mice after 6 or 12 wk western diet (**E**, n=8–12 per group). Scale bar=100 µm. *P<0.05. αBCR indicates anti-B cell receptor; B^tg^, B cell transgenic; CPM, counts per minute; FO, follicular; MZ, marginal zone 1; T1, transitional stage 1; T2, transitional stage 2; and WT, wild-type.

Overexpression of FcγRIIb on B cells, mimicking the endogenous upregulation that occurs during B cell activation, was shown to protect mice in models of arthritis and SLE.^13,29^ We, therefore, designed experiments to examine the effect of B cell FcγRIIb overexpression on atherosclerosis using this previously described FcγRIIb-B^tg^ mouse. The transgene results in specifically elevated FcγRIIb on B cells but not myeloid (CD11b^+^) cells (Figure 1A). We confirmed the functional impact of this by measuring the response to B cell activation in vitro with anti–B cell receptor (anti-IgM) antibodies with or without the FcγRIIb-recruiting Fc portion. The proliferation in response to anti-IgM IgG antibodies was severely attenuated in B cells from B^tg^ *Apoe*^−/−^ mice. When anti-IgM Fab’ fragments (lacking Fc receptor-binding capacity) were used, there was a much smaller difference between WT and B^tg^ B cells (Figure 1B), with the residual difference potentially caused by enhanced levels of apoptosis in B^tg^ B cells. Indeed, in vivo, B^tg^ mice have lower proportions of follicular B cells but similar proportions of other B-2 cell subsets (Figure 1C) and express elevated levels of proapoptotic Fas (Figure IIA in the online-only Data Supplement).

We first analyzed the impact on atherosclerosis using BM transplant from B^tg^ or nontransgenic littermate control mice into irradiated *Ldlr*^−/−^ mice. Atherosclerotic plaque size was reduced in *Ldlr*^−/−^/B^tg^ mice compared with *Ldlr*^−/−^/WT mice (Figure 1D). Total cholesterol, body weights, and circulating monocyte numbers were not different between groups (Figure IIB through IID in the online-only Data Supplement). Using the same *Ldlr*^−/−^ chimeric model, we did not observe any impact on atherosclerosis using mice with FcγRIIb overexpression driven by the macrophage-specific CD68 promoter^29^ (Figure IIE through IIH in the online-only Data Supplement), suggesting a more influential role for FcγRIIb-mediated control of B cells on atherosclerosis. Based on these results, we created B^tg^ *Apoe*^−/−^ mice and analyzed the impact of western diet–induced atherosclerosis in male mice. Atherosclerosis in the aortic root was similar after 6 weeks but significantly reduced in B^tg^ *Apoe*^−/−^ mice after 12 weeks of western diet (Figure 1E; Figure IIIA in the online-only Data Supplement). Again, there were no significant differences in total cholesterol, body weights, or circulating monocytes between groups (Figure IIIB through IIID in the online-only Data Supplement). There were no significant differences in spleen and BM cellularity between control and B^tg^ mice (Figure IIIE and IIIF in the online-only Data Supplement). Further analysis of the atherosclerotic plaques revealed that there were no significant differences in key plaque components such as macrophages, smooth muscle cells, necrotic core, or collagen (Figure IIIG through IIIJ in the online-only Data Supplement).

### B Cell Overexpression of FcγRIIb Reduces GC-Dependent Antibody Responses

**Figure 2.**
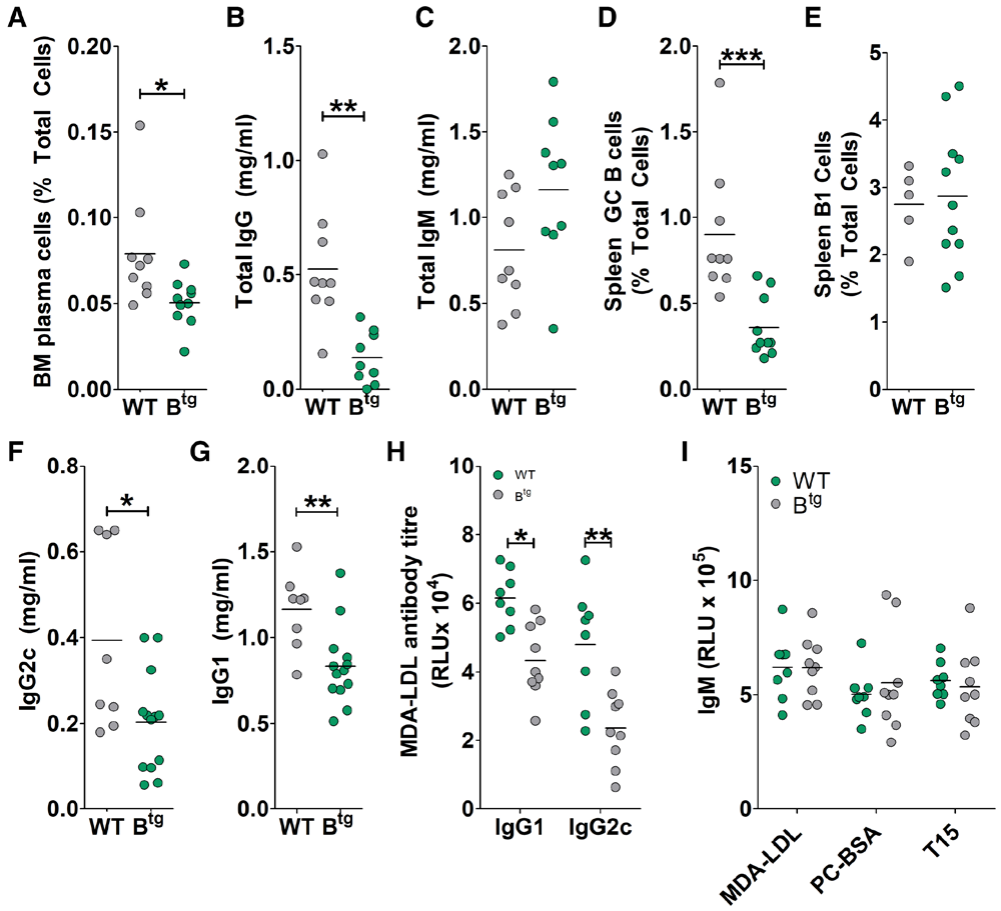
Atherosclerotic B cell transgenic (B^tg^) mice display reduced IgG antibody responses. Antibody-producing B cell subsets and serum antibody levels were quantified (by flow cytometry and ELISA, respectively) in tissues from western diet–fed *Apoe*^−/−^ or B^tg^ *Apoe*^−/−^ mice. **A**, Bone marrow (BM) plasma cells (B220^−^ CD138^hi^). Data from 1 *Apoe*^−/−^ mouse were excluded during data collection. **B** and **C**, Total serum IgG and IgM levels. **D**, Spleen germinal center (GC) B cell levels (CXCR5^+^ GL7^+^). **E**, Spleen B-1 cell levels (CD19^+^ B220^+/lo^ IgM^+^ CD43^+^ CD23^−^). **F** and **G**, Total serum IgG2c and IgG1. Data from 1 *Apoe*^−/−^ mouse were excluded during data collection. **H**, Serum MDA-LDL-binding IgG2c or IgG1. Data from 1 *Apoe*^−/−^ mouse were excluded during data collection. **I**, IgM antibodies to MDA-BSA, PC-BSA, or the T15 idiotype (AB1-2 antibodies). *P<0.05, **P<0.01, ***P<0.005. BSA indicates bovine serum albumin; LDL, low-density lipoprotein; MDA, malondialdehyde; PC, phosphorylcholine RLU, relative light units; and WT, wild-type.

B cells regulate atherosclerosis via multiple mechanisms (reviewed in Sage et al^2^), including regulation of T cells,^6,7,22,24,30^ via the cytokine BAFF^31^ or via antibody responses.^27,32,33^ We, therefore, analyzed whether any of these mechanisms could explain the impacts on atherosclerosis in the B^tg^ models. Previously, B cell depletion strategies led to reduced CD3^+^ staining in plaques and reduced T cell activation.^6,24^ Here, plaque CD3^+^ T cell levels were diminished in *Ldlr*^−/−^/B^tg^ mice but not in B^tg^ *Apoe*^−/−^ mice (Figure IVA and IVB in the online-only Data Supplement), despite a significant reduction in activated effector memory T cells (T_em_) in the spleen of both *Ldlr*^−/−^/B^tg^ and B^tg^ *Apoe*^−/−^ mice compared with their respective controls (Figure IVC and IVD in the online-only Data Supplement). We recently found an important protective role for BAFF in atherosclerosis, which we suggest acted via macrophage-expressed TACI (transmembrane activator and CAML interactor)^31^; this provides an alternative mechanism for the protective effect of B cell depletion, which results in high BAFF levels.^30^ In B^tg^ mice, BAFF levels were lower than in control mice (Figure IVE in the online-only Data Supplement). We then turned to the humoral response. A major phenotype of these mice is decreased plasma cell survival,^13^ which prompted us to analyze the humoral responses thought to play a role in atherosclerosis.^2^ Proportions of BM plasma cells were significantly reduced in B^tg^ *Apoe*^−/−^ mice compared with control littermates (Figure 2A). In agreement with this, total IgG levels in serum were decreased in B^tg^ *Apoe*^−/−^ mice (Figure 2B) and *Ldlr*^−/−^/B^tg^ mice (Figure IVF in the online-only Data Supplement) compared with respective controls, whereas total IgM levels were not affected (Figure 2C; Figure IVG in the online-only Data Supplement). FcγRIIb also directly inhibits GC B cells (by inhibiting BCR [B cell receptor] signaling^26^), which produce the class-switched antibody response and B-1 cells (by promoting apoptosis^14^), which mainly produce IgM. In accordance with the antibody data, GC B cells were reduced in B^tg^ *Apoe*^−/−^ mice compared with *Apoe*^−/−^ controls, whereas proportions of B-1 cells were not different (Figure 2D through 2E). The specific isotypes IgG1 and IgG2c are largely GC-dependent and known to be increased in atherosclerotic mice.^18^ The levels of both IgG1 and IgG2c were reduced in B^tg^ *Apoe*^−/−^ mice (Figure 2F and 2G). We also found similar reductions in MDA (malondialdehyde)-LDL-binding IgG1 and IgG2c (Figure 2H). In contrast, there were no differences in levels of IgM with specificities associated with B-1 cells, including MDA-LDL, antiphosphorylcholine, or the specific antiphosphorylcholine T15 clonotypic antibodies (Figure 2I). We also detected the same reductions in total and MDA-LDL IgG2c in *Ldlr*^−/−^/B^tg^ mouse serums, without alteration of IgG1 and IgM levels (Figure IVH through IVJ in the online-only Data Supplement). We concluded that B cell FcγRIIb–driven reduction in atherosclerosis was associated with reductions in systemic CD4^+^ T cell responses and IgG2c class-switched antibody responses.

### Selective Decrease of FcγRIIb in GC B Cells Increases Atherosclerosis

**Figure 3.**
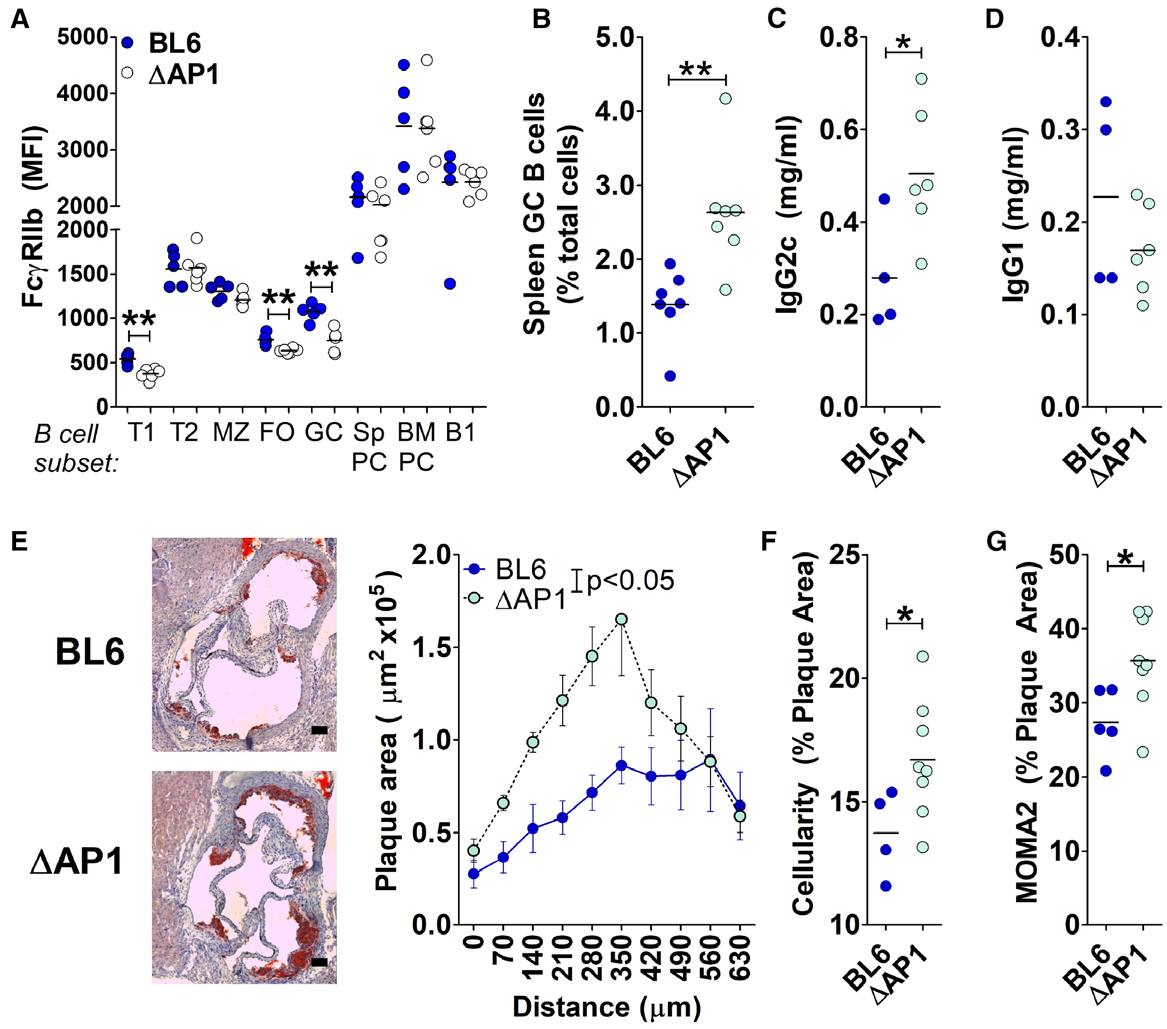
Decreased FcγRIIb (Fcγ receptor IIb) expression on germinal center (GC) B cells in FcγRIIb^ΔAP-1^ male mice enhances GC responses and increases atherosclerosis. **A**, Mean fluorescence intensity (MFI) of anti-FcγRIIb staining on B cells subsets (Figure I in the online-only Data Supplement) in *Apoe*^−/−^ or *FcγRIIb*^*ΔAP-1*^ *Apoe*^−/−^ mice. **B**, Spleen GC B cell levels. **C** and **D**, Total serum IgG2c and IgG1. **E**, Atherosclerosis quantified using Oil Red O–stained aortic root cryosections from male Apoe^−/−^ or *FcγRIIb*^*ΔAP-1*^ *Apoe*^−/−^ mice after 6-wk western diet. N=6 to 8 per group. Scale bar=100 µm. **F**–**H**, Atherosclerotic plaque composition was analyzed for proportions of cells (**F**) or MOMA2^+^-stained area (**G**). A single outlier was excluded from the WT group in **F**. *P<0.05. AP1 indicates activating protein-1; BM, bone marrow; FO, follicular; MZ, marginal zone; Sp, spleen; T1, transitional stage 1; and T2, transitional stage 2.

The decrease in class-switched antibodies in B^tg^ mice could be because of FcγRIIb acting directly on GC B cells, on which it inhibits activation, and on plasma cells, on which it promotes apoptosis. We, therefore, sought out a way to separate the impact of FcγRIIb on GC B cells from its role on plasma cells. Inbred mouse strains as well as wild-type strains from around the world display distinct haplotypes at the FcγRIIb locus.^26^ The haplotype carried by New Zealand black mice lacks an AP-1 (activating protein-1)-binding site in the proximal promoter region and was reported to lead to defective upregulation of FcγRIIb specifically upon GC B cell recruitment.^26^ By knocking in the New Zealand black haplotype FcγRIIb promoter sequence into the C57BL/6 mouse genome, FcγRIIb^ΔAP-1^ mice display decreased FcγRIIb expression on GC B cells and consequently enhanced GC formation.^26^ Importantly, for our studies, FcγRIIb^ΔAP-1^ mice have a mild autoimmune phenotype with no overt clinical signs up to 14 months, with glomerular IgG deposition but no decrease in kidney function evident at that age.^26^ We crossed FcγRIIb^ΔAP-1^ mice to the *Apoe*^−/−^ background and confirmed comparatively reduced FcγRIIb levels on GC B cells in FcγRIIb^ΔAP-1^ compared with control (FcγRIIb^BL6^) littermates (Figure 3A). Some reduction was also detectable in transitional cells entering the spleen and on naive follicular cells; however, no difference was detected in marginal zone, B-1, or plasma cells, which all have higher expression levels than naïve B cells (Figure 3A), suggesting that the AP-1-binding site is not critical for upregulation in those cells. Indeed, the B cell compartment was grossly normal in FcγRIIb^ΔAP-1^ compared with WT controls, with no differences in proportions of different B cell subsets (Figure VA in the online-only Data Supplement). Atherosclerosis-prone FcγRIIb^ΔAP-1^ mice displayed a significant enhancement of GC B cells, follicular helper T cells (that participate in the GC reaction), and circulating IgG2c antibodies compared with FcγRIIb^BL6^ littermates (Figure 3B and 3C; Figure VB in the online-only Data Supplement). Serum IgG1 levels were not different (Figure 3D). In support of a proatherogenic role of enhanced GC responses, FcγRIIb^ΔAP-1^ mice had bigger atherosclerotic plaques compared with FcγRIIb^BL6^ littermates (Figure 3E). This was not associated with changes in plasma cholesterol, body weight, or circulating monocytes (Figure VC through VE in the online-only Data Supplement). Atherosclerotic plaques were more cellular and contained more macrophages (Figure 3F and 3G), whereas CD3^+^ T cell proportions were similar to control mice (Figure VF in the online-only Data Supplement). Taken together, GC-B cell intrinsic FcγRIIb levels control a proatherogenic response.

### IgG2c Induces TNF in FcγRIV-Expressing CD11c^+^ CD11b^+^ Cells

**Figure 4.**
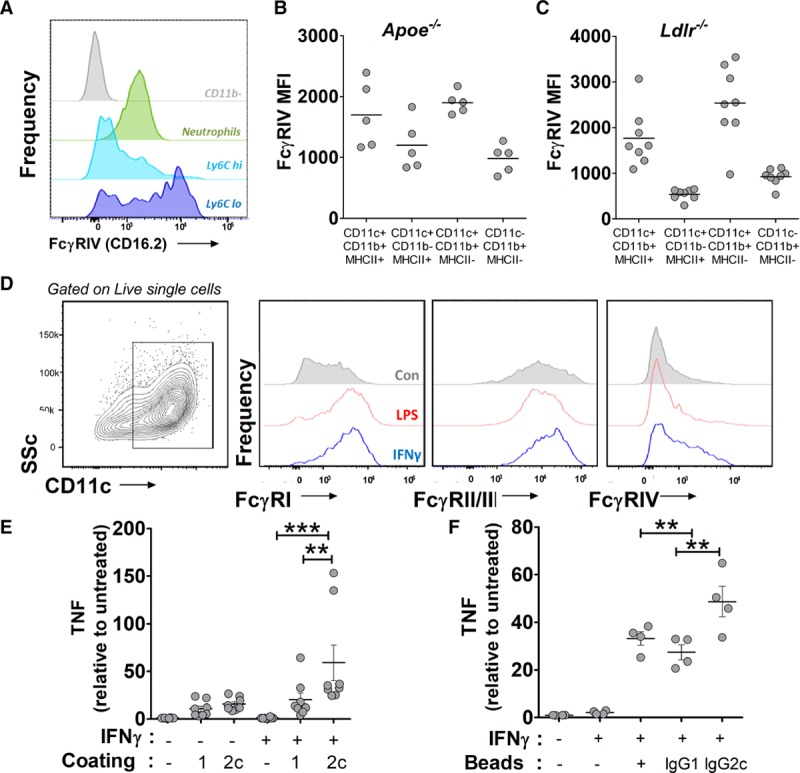
IgG2c induces TNF (tumor necrosis factor) production by FcγRIV-expressing CD11b^+^ CD11c^+^ cells. **A**, Histograms of anti-FcgRIV staining on mouse blood myeloid cell subsets. **B** and **C**, *Apoe*^−/−^ (**B**) or Ldlr^−/−^ (**C**) mouse aortas were analyzed by flow cytometry for FcyRIV mean fluorescent intensity (MFI) in myeloid cell subsets based on CD11b, CD11c, and MHCII expression. **D**, GM-CSF (granulocyte-monocyte colony stimulating factor)-induced CD11c^+^ CD11b^+^ MHCII^+^ cells were incubated with IFNγ (interferon-γ) for 24 h and then analyzed by flow cytometry for FcyRI, FcyRIIb/III, and FcgRIV. **E**, TNF production by GM-CSF–induced CD11c^+^ CD11b^+^ MHCII^+^ cells after culture for 48 h±IFNγ on uncoated, mouse IgG2c, or mouse IgG1-coated plates. Relative levels in 4 independent experiments. **F**, TNF production by IFNγ-pretreated GM-CSF-induced CD11c^+^ CD11b^+^ MHCII^+^ cells after culture for 48 h with uncoated, mouse IgG2c-coated, or mouse IgG1-coated beads. Relative levels in 2 independent experiments. *P<0.05, **P<0.01, ***P<0.005. GM-CSF indicates granulocyte-monocyte colony stimulating factor; MHCII, major histocompatibility complex II; LPS, lipopolysaccharide; and SSC, side scatter.

To understand the potential for IgG2c to directly regulate atherosclerosis, we analyzed the expression of its proinflammatory receptor FcγRIV (which is mainly expressed in myeloid cell subsets). FcγRIV was most highly expressed on Ly6C^lo^ monocytes in the blood, consistent with previous reports^34^ (Figure 4A). In the atherosclerotic mouse aorta, CD45^+^ CD11b^+^ CD11c^+^ cells (monocyte-derived macrophages/dendritic cells) expressed high levels of FcγRIV (Figure 4B and 4C). To investigate the response of these cells to IgG2c in vitro, we cultured CD11b^+^ CD11c^+^ MHCII^+^ cells using BM monocytes differentiated with GM-CSF (moDCs). moDCs upregulated FcγRIV in response to IFNγ, a known proatherosclerotic cytokine (Figure 4D). We then investigated the effect of mouse IgG2c and IgG1 antibodies as immune complexes on moDC activation, measured by TNF secretion, in 2 ways. First, we precoated culture plates with either IgG2c or IgG1 and then added moDCs in the presence of IFNγ. After 48 hours, IFNγ-treated moDCs alone produced low levels of TNF (Figure 4E). moDCs cultured in IgG2c-coated wells produced significantly more TNF than IgG1, supporting a more proinflammatory effect on these cells (Figure 4E). Next, we made antibody-coated particles (that could mimic antibody-coated oxLDL or necrotic debris in plaques) using polystyrene beads coated with IgG2c or IgG1. Coated beads (or control uncoated beads) were incubated with IFNγ-pretreated moDCs and TNF production quantified. Uncoated beads induced some TNF production above IFNγ-treated moDCs without beads (Figure 4F); however, IgG2c-coated beads produced increased TNF, whereas IgG1-coated beads did not (Figure 4F). These data support the notion that IgG2c antibodies could enhance inflammatory responses by plaque macrophages or dendritic cells.

### B cell FcγRIIb Overexpression in Female Mice Decreases IgM and Enhances Atherosclerosis

**Figure 5.**
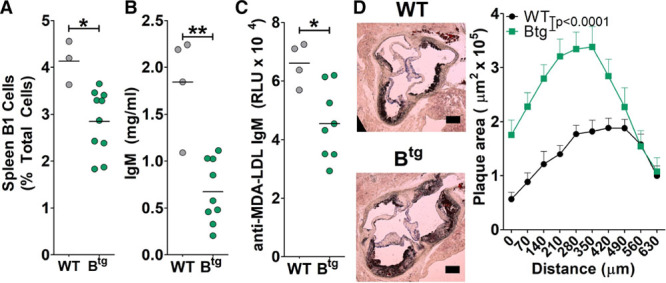
B cell FcγRIIb (Fcγ receptor IIb) expression levels are important for B-1-cell responses in female atherosclerotic mice. **A**–**C**, B-1-cell responses in tissues from western diet–fed female *Apoe*^−/−^ or B^tg^ *Apoe*^−/−^ mice. **A**, Spleen B-1 cell levels (CD19^+^ B220^+/lo^ IgM^+^ CD43^+^ CD23^−^). **B**, Total serum IgM levels. **C**, MDA-LDL (malondialdehyde-low-density lipoprotein)-binding IgM levels. **D**, Atherosclerosis quantified using oil red O–stained aortic root cryosections after 6-wk western diet. N=7 to 8 per group. Scale bar=100 µm. *P<0.05. B^tg^ indicates B cell transgenic; LDL, low-density lipoprotein; MDA, malondialdehyde; RLU, relative light units; and WT, wild-type.

We were initially surprised that IgM levels were not modulated in B^tg^ mice because FcγRIIb should also negatively regulate IgM^+^ plasma cells and has also been shown to promote B-1 cell apoptosis.^14^ Indeed, when we purified B-1 cells from B^tg^ *Apoe*^−/−^ and *Apoe*^−/−^ mice and incubated them in control or anti-FcγRIIb–coated plates, B^tg^ *Apoe*^−/−^ B cells were more prone to undergo apoptosis (Figure VIA in the online-only Data Supplement; assessed by Annexin V exposure; see Methods). This suggests that in vivo in male B^tg^ mice, the pool of IgM-secreting B-1 cells or other IgM-producing B cells was renewed rapidly enough from resting B cells to compensate for a shorter lifespan of IgM antigen-secreting cells. However, when we investigated female B^tg^ *Apoe*^−/−^ mice, we did observe a reduction in B-1 cells, total IgM levels, and MDA-LDL IgM levels (Figure 5A through 5C), with other B cell parameters similar to males (data not shown). Indeed, as the major observed difference between genders, the reduced IgM in females seems to be critical to the outcome of atherosclerotic plaque development because female B^tg^ *Apoe*^−/−^ had significantly enhanced atherosclerosis (Figure 5D) in contrast to the male counterparts (Figure 1) but similar to antibody-deficient Xbp1^cKO^ mice^27^ and B cell (and therefore antibody)–deficient mice.^35^ The plaques of B^tg^ *Apoe*^−/−^ female mice had larger necrotic cores (Figure VIB in the online-only Data Supplement). Therefore, a combined deficiency in IgG and IgM may provide an explanation for the accelerated atherosclerosis in female mice in this setting.

### Male and Female *Apoe*^−/−^ Mice Display Different B-1 Cell Dynamics In Vivo and In Vitro

**Figure 6.**
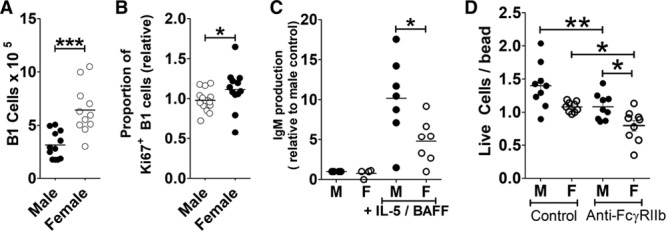
B-1 cells from female Apoe^−/−^ mice are more susceptible to apoptosis. **A**, Spleen B-1 cell levels in male and female *Apoe*^−/−^ mice (CD19^+^ B220^+/lo^ IgM^+^ CD43^+^ CD23^−^; pooled data from 4 experiments). **B**, Proportion of spleen B-1 cells staining positive for Ki67 (relative to males, 4 experiments pooled). **C**, IgM production by purified B-1 cells from male or female *Apoe*^−/−^ mice after 72 h with IL-5 and BAFF (B cell–activating factor; relative to males, 4 pooled experiments in at least duplicate). **D**, B-1 cells purified from peritoneum of male or female *Apoe*^−/−^ mice were cultured with or without anti-FcγRIIB (2.4G2 antibody) and then stained with annexin V and propidium iodide. Absolute numbers of remaining live cells (AnnV^−^ PI^−^) was quantified by flow cytometry using beads for normalization. *P<0.05, **P<0.01, ***P<0.005.

We investigated several possibilities that could explain the sex disparity. There was no sex-specific difference in FcγRIIb overexpression levels in B^tg^ mice or in baseline FcγRIIb levels in control littermates on B-1 cells (Figure VIC in the online-only Data Supplement). We did observe, by analyzing past data from multiple experiments and strains on the *Apoe*^−/−^ background, that female *Apoe*^−/−^ mice had higher B-1 cell numbers in the spleen (Figure 6A). A higher proportion of female B-1 cells stained positive for the proliferation marker Ki67 than in male B-1 cells in the spleen (Figure 6B), where B-1 cells migrate to self-renew or differentiate into plasma cells.^21,36^ Despite the difference in splenic B-1 cell levels between males and females, which was highly reproducible, there were no consistent differences in total or oxidation-specific epitope–specific IgM levels or IgM-producing plasma cells between male and female *Apoe*^−/−^ mice (data not shown). We hypothesized that a larger supply of B-1 cells is required to maintain similar steady-state levels of IgM in females than in males, at least in the context of the high demand expected in atherosclerotic mice, where splenic B-1 cells and IgM levels expand compared with WT mice. This could then provide an explanation why in B^tg^ mice, where there is even further pressure on B-1 cell numbers, a difference in B-1 cells and IgM levels is seen in females not males. This higher turnover in females could be explained by a higher clearance/degradation rate of IgM or a shorter lifespan of B-1 plasma cells. First, IgM clearance rate was investigated by transferring serum with antibodies of the “a” heavy chain isotype (from Balb/c mice) into *Apoe*^−/−^ mice, which express the “b” heavy chain isotype, allowing quantification of injected IgM^a^ by ELISA. The clearance of IgM^a^ was tracked over 48 hours, but we did not observe a difference in half-life between male and female *Apoe*^−/−^ mice (Figure VID in the online-only Data Supplement). Second, in vitro, we assessed the ability of male and female B-1 cells to form IgM-secreting plasma cells in response to IL-5 and BAFF. We found a decreased ability of female B-1 cells to produce IgM compared with males (Figure 6C and 6D). Female B-1 cells showed enhanced susceptibility to apoptosis, with fewer female B-1 cells than male B-1 cells alive after overnight culture (staining negative for Annexin V and propidium iodide; Figure 6E). Elispot assays for IgM demonstrated that female IgM antigen-secreting cell produce similar amounts of IgM per cell (spot size; Figure VIE in the online-only Data Supplement).

Female FcγRIIb^ΔAP-1^ mice were also different from male counterparts. Despite a similar decrease (to that seen in males) in FcγRIIb levels on GC B cells of female FcγRIIb^ΔAP-1^ mice compared with control littermates (Figure VIIA in the online-only Data Supplement), there was no difference in proportions of GC B cells (Figure VIIB in the online-only Data Supplement), total IgG2c (Figure VIIC in the online-only Data Supplement), or total IgM (Figure VIID in the online-only Data Supplement), and consequently no difference in atherosclerosis (Figure VIIE in the online-only Data Supplement). Female *Apoe*^−/−^ mice had higher proportions of GC B cells (compare Figure 3B and Figure VIIB in the online-only Data Supplement), follicular helper T (Figure VIIF in the online-only Data Supplement), increased anti-dsDNA autoantibodies (Figure VIIG in the online-only Data Supplement), and increased IgG2c antibodies (Figure VIIH in the online-only Data Supplement) compared with males, suggesting an already enhanced autoimmune susceptibility that may not be increased so easily as in the males by the FcγRIIb mutation.

## Discussion

Systemic and local inflammation is increasingly accepted as a major independent driver of atherosclerotic cardiovascular disease. Inflammation can induce and accelerate autoimmune responses that contribute to local tissue damage and prevent resolution.^37^ Here, we report the novel finding that B cell–intrinsic control of autoimmune GC responses by the IgG receptor FcγRIIb limits atherosclerotic plaque development in mice. Although a role for FcγRIIB has been previously reported, the influence and mechanism, and in particular the main cell type expressing FcγRIIB, are not understood. Our results support a pathogenic role for GC-dependent responses, and we propose that IgG2c class switching may be a key pathogenic pathway. In addition, (1) we reveal a potential explanation for the opposite effects on atherosclerosis observed in *Fcgr2b*^−/−^ mice and other models modulating both B-1 and B-2 cells and (2) provide further support for the paradigm that autoimmune responses in mouse atherosclerosis, like in humans, are sex-dependent.

Our study is the first to reveal the importance of B cell FcγRIIb in experimental atherosclerosis. In addition to plasma cell responses, FcγRIIb also controls the GC response and is especially important in limiting access by autoreactive B cells. Data from both models suggest that GC-dependent responses may promote atherosclerosis, consistent with previous data implicating the GC as pathogenic.^25,38,39^ Overexpression of FcγRIIb led to a decrease in GC B cells and GC-dependent IgG isotypes (Figure 2). More specifically, limiting FcγRIIb upregulation on GC B cells but not plasma cells, B-1 cells or marginal zone B cells led to increased atherosclerosis, associated with an enhanced GC response (Figure 3). Crucially, in FcγRIIb^ΔAP-1^ mice, we were not able to detect differences in effector T cell responses (except GC-promoting follicular helper T) and only observed an increase in IgG2c antibodies, not IgG1. The latter effect may reflect the relatively short duration of the experiments, Th2-dependent IgG1 responses being more prevalent after prolonged high-fat diet.^40^ IgG2c is known to have a particularly potent proinflammatory Fc receptor–binding profile.^34^ The Fc portion of IgG2c has a much higher bias for binding to activating FcγRIV than inhibitory FcγRIIb. In vitro, FcγRIV binding stimulates proinflammatory cytokine production and T cell stimulatory function by macrophages and dendritic cells.^34,41^ Here, we found that moDCs like those found in plaques produced high levels of TNF in response to IgG2c when stimulated to upregulate FcγRIV with IFNγ (Figure 4). In agreement with our studies, atherosclerotic macrophages/dendritic cells express FcγRIV,^42,43^ and the activatory:inhibitory FcγR ratio of inflammatory monocytes is increased in western diet compared with chow conditions (analysis of data from Kim et al^43^). We thus suggest that GC-derived antibodies such as IgG2c may have direct proatherogenic effects on plaque (or inflamed vessel) innate immune cells.

The human equivalent of IgG2c-FcγRIV interactions is thought to be IgG1-FcγRIIIA.^11^ Recently, a subset of human monocytes expressing the surface marker SLAN (6-sulfo N-acetyllactosamine; SLAN monocytes) were found to express FcγRIIIA (CD16), be the most responsive to immune complexes, and make high levels of TNF in response to human IgG1.^44^ This same subset is most expanded in patients with cardiovascular disease.^45^ Human IgG levels have been positively associated with cardiovascular risk, although not in all studies.^46^ As exemplified by this study and others,^27,33,39^ antibodies have multiple relevant functions, and further work is needed to understand and isolate these individual influences.

Multiple studies have investigated atherosclerosis using *Fcg2rb*^−/−^ mice. In summary, although the B cell phenotype of increased IgG, and in most cases increased IgM, is consistent across studies, other factors seem to be inconsistent and affected by the background strain of the mice, timings, and methodology. Interestingly, although Merched et al^20^ observed a big increase in IgG, they did not observe differences in IgM levels, which based on our studies would be consistent with the increased atherosclerosis they reported. Multiple explanations for the effects on atherosclerosis observed in *Fcgr2b*^−/−^ mice are possible since several anti-inflammatory pathways (increased Treg, increased Th2 cells, an M1 to M2 switch in macrophages, cytokine production by macrophages) and pathogenic pathways (autoantibodies, increased CCL2, increased T cell infiltration, more IL-17 and IL-23, increased FcγRIII on macrophages) are implicated across the various studies. Ng et al^47^ found a similar reduction in atherosclerosis in both standard and BM transplant experiments, suggesting that hematopoietic cell FcγRIIb deficiency was a key player in that study. Sex was important in 1 study using *Fcgr2b*^−/−^ mice,^18^ as observed in our study, but a similar effect was observed in both genders in another study.^47^ Since the anti-inflammatory pathways listed above are not B cell intrinsic, they are likely to have different sex dependency. For example, IFNγ deficiency only reduced atherosclerosis in male but not in female mice.^48^ Here, we found that modulation of IgM levels occurred only in female B^tg^ mice despite a similar effect in both males and females on FcγRIIB overexpression. FcγRIIb appeared less important for the GC response in females, which might be explained by the already increased GC levels and autoantibodies, consistent with the enhanced susceptibility to autoimmunity of females, both in mice and in humans.^49^ Both estrogen and testosterone have been implicated in regulating B cells. For example, a recent study demonstrated that testosterone regulation of BAFF production in the spleen is important in regulating the B cell compartment,^50^ and a complement-independent natural antibody pathway for bacterial clearance is driven by estrogen and is only effective in females.^51^ In humans, Frostegård et al^52^ report higher antiphosphorylcholine IgM levels in females in multiple cohorts. Further studies investigating the differences between male and female B cell systems, in particular, B-1 cells, will be necessary to better understand the sex-dependent regulation of B cell responses in atherosclerosis.

In summary, our studies make further strides toward understanding B cell regulation of atherosclerosis and support the pathogenic involvement of an active, Th1-polarized GC-dependent response controlled by FcγRIIb levels. Our data further support an important influence of B-1 cell–derived IgM but also now suggest the relative importance of these responses is sex dependent. Given the enhanced adaptive response associated with vulnerable plaques,^53^ targeting such responses may be a future therapeutic strategy.

## Acknowledgments

This research was supported by the Cambridge NIHR BRC (National Institute for Health Research Biomedical Research Center) Cell Phenotyping Hub. We also thank Maria Ozsvar-Kozma for expert technical assistance.

## Sources of Funding

This work was supported by British Heart Foundation grants to A.P. Sage (FS/15/57/31557) and Z. Mallat.

## Disclosures

None.

## Supplementary Material

## References

[1] Palinski W, Tangirala RK, Miller E, Young SG, Witztum JL (1995). Increased autoantibody titers against epitopes of oxidized LDL in LDL receptor-deficient mice with increased atherosclerosis.. Arterioscler Thromb Vasc Biol.

[2] Sage AP, Tsiantoulas D, Binder CJ, Mallat Z (2019). The role of B cells in atherosclerosis.. Nat Rev Cardiol.

[3] Binder CJ, Papac-Milicevic N, Witztum JL (2016). Innate sensing of oxidation-specific epitopes in health and disease.. Nat Rev Immunol.

[4] Bruce IN (2005). ‘Not only.but also’: factors that contribute to accelerated atherosclerosis and premature coronary heart disease in systemic lupus erythematosus.. Rheumatology (Oxford).

[5] Goodnow CC, Sprent J, Fazekas de St Groth B, Vinuesa CG (2005). Cellular and genetic mechanisms of self tolerance and autoimmunity.. Nature.

[6] Ait-Oufella H, Herbin O, Bouaziz JD, Binder CJ, Uyttenhove C, Laurans L, Taleb S, Van Vré E, Esposito B, Vilar J, Sirvent J, Van Snick J, Tedgui A, Tedder TF, Mallat Z (2010). B cell depletion reduces the development of atherosclerosis in mice.. J Exp Med.

[7] Kyaw T, Tay C, Khan A, Dumouchel V, Cao A, To K, Kehry M, Dunn R, Agrotis A, Tipping P, Bobik A, Toh BH (2010). Conventional B2 B cell depletion ameliorates whereas its adoptive transfer aggravates atherosclerosis.. J Immunol.

[8] Zouggari Y, Ait-Oufella H, Bonnin P (2013). B lymphocytes trigger monocyte mobilization and impair heart function after acute myocardial infarction.. Nat Med.

[9] Schaheen B, Downs EA, Serbulea V, Almenara CC, Spinosa M, Su G, Zhao Y, Srikakulapu P, Butts C, McNamara CA, Leitinger N, Upchurch GR, Meher AK, Ailawadi G (2016). B-cell depletion promotes aortic infiltration of immunosuppressive cells and is protective of experimental aortic aneurysm.. Arterioscler Thromb Vasc Biol.

[10] Chan CT, Sobey CG, Lieu M (2015). Obligatory role for b cells in the development of angiotensin ii-dependent hypertension.. Hypertension.

[11] Nimmerjahn F, Ravetch JV (2008). Fcgamma receptors as regulators of immune responses.. Nat Rev Immunol.

[12] Smith KG, Clatworthy MR (2010). FcgammaRIIB in autoimmunity and infection: evolutionary and therapeutic implications.. Nat Rev Immunol.

[13] Xiang Z, Cutler AJ, Brownlie RJ, Fairfax K, Lawlor KE, Severinson E, Walker EU, Manz RA, Tarlinton DM, Smith KG (2007). FcgammaRIIb controls bone marrow plasma cell persistence and apoptosis.. Nat Immunol.

[14] Amezcua Vesely MC, Schwartz M, Bermejo DA, Montes CL, Cautivo KM, Kalergis AM, Rawlings DJ, Acosta-Rodríguez EV, Gruppi A (2012). FcγRIIb and BAFF differentially regulate peritoneal B1 cell survival.. J Immunol.

[15] Jonsson S, Sveinbjornsson G, de Lapuente Portilla AL (2017). Identification of sequence variants influencing immunoglobulin levels.. Nat Genet.

[16] Kyogoku C, Dijstelbloem HM, Tsuchiya N, Hatta Y, Kato H, Yamaguchi A, Fukazawa T, Jansen MD, Hashimoto H, van de Winkel JG, Kallenberg CG, Tokunaga K (2002). Fcgamma receptor gene polymorphisms in Japanese patients with systemic lupus erythematosus: contribution of FCGR2B to genetic susceptibility.. Arthritis Rheum.

[17] Zhao M, Wigren M, Dunér P, Kolbus D, Olofsson KE, Björkbacka H, Nilsson J, Fredrikson GN (2010). FcgammaRIIB inhibits the development of atherosclerosis in low-density lipoprotein receptor-deficient mice.. J Immunol.

[18] Mendez-Fernandez YV, Stevenson BG, Diehl CJ, Braun NA, Wade NS, Covarrubias R, van Leuven S, Witztum JL, Major AS (2011). The inhibitory FcγRIIb modulates the inflammatory response and influences atherosclerosis in male apoE(-/-) mice.. Atherosclerosis.

[19] Harmon EY, Fronhofer V, Keller RS, Feustel PJ, Zhu X, Xu H, Avram D, Jones DM, Nagarajan S, Lennartz MR (2014). Anti-inflammatory immune skewing is atheroprotective: Apoe−/−FcγRIIb−/− mice develop fibrous carotid plaques.. J Am Heart Assoc.

[20] Merched AJ, Daret D, Li L, Franzl N, Sauvage-Merched M (2016). Specific autoantigens in experimental autoimmunity-associated atherosclerosis.. FASEB J.

[21] Kyaw T, Tay C, Krishnamurthi S, Kanellakis P, Agrotis A, Tipping P, Bobik A, Toh BH (2011). B1a B lymphocytes are atheroprotective by secreting natural IgM that increases IgM deposits and reduces necrotic cores in atherosclerotic lesions.. Circ Res.

[22] Nus M, Sage AP, Lu Y (2017). Marginal zone B cells control the response of follicular helper T cells to a high-cholesterol diet.. Nat Med.

[23] Strom AC, Cross AJ, Cole JE, Blair PA, Leib C, Goddard ME, Rosser EC, Park I, Hultgårdh Nilsson A, Nilsson J, Mauri C, Monaco C (2015). B regulatory cells are increased in hypercholesterolaemic mice and protect from lesion development via IL-10.. Thromb Haemost.

[24] Sage AP, Tsiantoulas D, Baker L, Harrison J, Masters L, Murphy D, Loinard C, Binder CJ, Mallat Z (2012). BAFF receptor deficiency reduces the development of atherosclerosis in mice–brief report.. Arterioscler Thromb Vasc Biol.

[25] Tay C, Liu YH, Kanellakis P, Kallies A, Li Y, Cao A, Hosseini H, Tipping P, Toh BH, Bobik A, Kyaw T (2018). Follicular B cells promote atherosclerosis via T Cell-mediated differentiation into plasma cells and secreting pathogenic immunoglobulin G.. Arterioscler Thromb Vasc Biol.

[26] Espéli M, Clatworthy MR, Bökers S, Lawlor KE, Cutler AJ, Köntgen F, Lyons PA, Smith KG (2012). Analysis of a wild mouse promoter variant reveals a novel role for FcγRIIb in the control of the germinal center and autoimmunity.. J Exp Med.

[27] Sage AP, Nus M, Bagchi Chakraborty J, Tsiantoulas D, Newland SA, Finigan AJ, Masters L, Binder CJ, Mallat Z (2017). X-box binding protein-1 dependent plasma cell responses limit the development of atherosclerosis.. Circ Res.

[28] Chou MY, Fogelstrand L, Hartvigsen K, Hansen LF, Woelkers D, Shaw PX, Choi J, Perkmann T, Bäckhed F, Miller YI, Hörkkö S, Corr M, Witztum JL, Binder CJ (2009). Oxidation-specific epitopes are dominant targets of innate natural antibodies in mice and humans.. J Clin Invest.

[29] Brownlie RJ, Lawlor KE, Niederer HA, Cutler AJ, Xiang Z, Clatworthy MR, Floto RA, Greaves DR, Lyons PA, Smith KG (2008). Distinct cell-specific control of autoimmunity and infection by FcgammaRIIb.. J Exp Med.

[30] Ponnuswamy P, Joffre J, Herbin O, Esposito B, Laurans L, Binder CJ, Tedder TF, Zeboudj L, Loyer X, Giraud A, Zhang Y, Tedgui A, Mallat Z, Ait-Oufella H (2017). Angiotensin II synergizes with BAFF to promote atheroprotective regulatory B cells.. Sci Rep.

[31] Tsiantoulas D, Sage AP, Göderle L, Ozsvar-Kozma M, Murphy D, Porsch F, Pasterkamp G, Menche J, Schneider P, Mallat Z, Binder CJ (2018). B cell-activating factor neutralization aggravates atherosclerosis.. Circulation.

[32] Kyaw T, Tipping P, Bobik A, Toh BH (2012). Protective role of natural IgM-producing B1a cells in atherosclerosis.. Trends Cardiovasc Med.

[33] Tsiantoulas D, Bot I, Ozsvar-Kozma M, Göderle L, Perkmann T, Hartvigsen K, Conrad DH, Kuiper J, Mallat Z, Binder CJ (2017). Increased plasma IgE accelerate atherosclerosis in secreted IgM deficiency.. Circ Res.

[34] Nimmerjahn F, Lux A, Albert H, Woigk M, Lehmann C, Dudziak D, Smith P, Ravetch JV (2010). FcγRIV deletion reveals its central role for IgG2a and IgG2b activity in vivo.. Proc Natl Acad Sci USA.

[35] Major AS, Fazio S, Linton MF (2002). B-lymphocyte deficiency increases atherosclerosis in LDL receptor-null mice.. Arterioscler Thromb Vasc Biol.

[36] Baumgarth N (2016). B-1 Cell Heterogeneity and the regulation of natural and antigen-induced IgM production.. Front Immunol.

[37] Libby P, Tabas I, Fredman G, Fisher EA (2014). Inflammation and its resolution as determinants of acute coronary syndromes.. Circ Res.

[38] Clement M, Guedj K, Andreata F (2015). Control of the T follicular helper-germinal center B-cell axis by CD8^+^ regulatory T cells limits atherosclerosis and tertiary lymphoid organ development.. Circulation.

[39] Centa M, Jin H, Hofste L Germinal center-derived antibodies promote atherosclerosis plaque size and stability [published online Mar 21, 2019].. Circulation.

[40] Zhou X, Paulsson G, Stemme S, Hansson GK (1998). Hypercholesterolemia is associated with a T helper (Th) 1/Th2 switch of the autoimmune response in atherosclerotic apo E-knockout mice.. J Clin Invest.

[41] Lehmann CHK, Baranska A, Heidkamp GF (2017). DC subset-specific induction of T cell responses upon antigen uptake via Fcγ receptors in vivo.. J Exp Med.

[42] Cochain C, Vafadarnejad E, Arampatzi P, Pelisek J, Winkels H, Ley K, Wolf D, Saliba AE, Zernecke A (2018). Single-cell RNA-seq reveals the transcriptional landscape and heterogeneity of aortic macrophages in murine atherosclerosis.. Circ Res.

[43] Kim K, Shim D, Lee JS (2018). Transcriptome analysis reveals nonfoamy rather than foamy plaque macrophages are proinflammatory in atherosclerotic murine models.. Circ Res.

[44] Olaru F, Dobel T, Lonsdorf AS, Oehrl S, Maas M, Enk AH, Schmitz M, Grone EF, Grone HJ, Schakel K (2018). Intracapillary immune complexes recruit and activate slan-expressing cd16+ monocytes in human lupus nephritis.. JCI Insight.

[45] Hamers AAJ, Dinh HQ, Thomas GD, Marcovecchio P, Blatchley A, Nakao CS, Kim C, McSkimming C, Taylor AM, Nguyen AT, McNamara CA, Hedrick CC (2019). Human monocyte heterogeneity as revealed by high-dimensional mass cytometry.. Arterioscler Thromb Vasc Biol.

[46] Iseme RA, McEvoy M, Kelly B, Agnew L, Walker FR, Handley T, Oldmeadow C, Attia J, Boyle M (2017). A role for autoantibodies in atherogenesis.. Cardiovasc Res.

[47] Ng HP, Zhu X, Harmon EY, Lennartz MR, Nagarajan S (2015). Reduced atherosclerosis in apoE-inhibitory FcγRIIb-deficient mice is associated with increased anti-inflammatory responses by T cells and macrophages.. Arterioscler Thromb Vasc Biol.

[48] Whitman SC, Ravisankar P, Daugherty A (2002). IFN-gamma deficiency exerts gender-specific effects on atherogenesis in apolipoprotein E-/- mice.. J Interferon Cytokine Res.

[49] Singh N, Johnstone DB, Martin KA, Tempera I, Kaplan MJ, Denny MF (2016). Alterations in nuclear structure promote lupus autoimmunity in a mouse model.. Dis Model Mech.

[50] Wilhelmson AS, Lantero Rodriguez M, Stubelius A (2018). Testosterone is an endogenous regulator of BAFF and splenic B cell number.. Nat Commun.

[51] Zeng Z, Surewaard BGJ, Wong CHY, Guettler C, Petri B, Burkhard R, Wyss M, Le Moual H, Devinney R, Thompson GC, Blackwood J, Joffe AR, McCoy KD, Jenne CN, Kubes P (2018). Sex-hormone-driven innate antibodies protect females and infants against EPEC infection.. Nat Immunol.

[52] Fiskesund R, Stegmayr B, Hallmans G, Vikström M, Weinehall L, de Faire U, Frostegård J (2010). Low levels of antibodies against phosphorylcholine predict development of stroke in a population-based study from northern Sweden.. Stroke.

[53] van Dijk RA, Duinisveld AJ, Schaapherder AF, Mulder-Stapel A, Hamming JF, Kuiper J, de Boer OJ, van der Wal AC, Kolodgie FD, Virmani R, Lindeman JH (2015). A change in inflammatory footprint precedes plaque instability: a systematic evaluation of cellular aspects of the adaptive immune response in human atherosclerosis.. J Am Heart Assoc.

