# Supplementary material for: B Cell Fcγ Receptor IIb Modulates Atherosclerosis in Male and Female Mice by Controlling Adaptive Germinal Center and Innate B-1-Cell Responses
Source: Arterioscler Thromb Vasc Biol. 2019 May 16;39(7):1379–89. doi: 10.1161/ATVBAHA.118.312272 (PMC6636804; doi:10.1161/ATVBAHA.118.312272)
Supplement: Supplementary file 1 [file atv-39-1379-s001.pdf]

Chakraborty et al

B cell FcγRIIb and Atherosclerosis

Major Resources Tables

Animals (in vivo studies)

Strain	Vendor or Source	Background Strain	Sex
FcγRIIb^{Btg}	Authors	C57BL6	Both
FcγRIIb^{Mtg}	Authors	C57BL6	Males
FcγRIIb$^{\Delta AP\text{-}1}$	Authors	C57BL6	Both
Ldlr$^{-/-}$	Jackson Labs	C57BL6	Male
Apoe$^{-/-}$	Charles River	C57BL6	Both

Animal breeding

Animal breeding for experiments is detailed in methods. *Ldlr*$^{-/-}$ and *Apoe*$^{-/-}$ strains were maintained by homozygous x homozygous breeding.

Antibodies

See supplemental table I.
