# Supplementary figures and images for: B Cell Fcγ Receptor IIb Modulates Atherosclerosis in Male and Female Mice by Controlling Adaptive Germinal Center and Innate B-1-Cell Responses
Source: Arterioscler Thromb Vasc Biol. 2019 May 16;39(7):1379–89. doi: 10.1161/ATVBAHA.118.312272 (PMC6636804; doi:10.1161/ATVBAHA.118.312272)

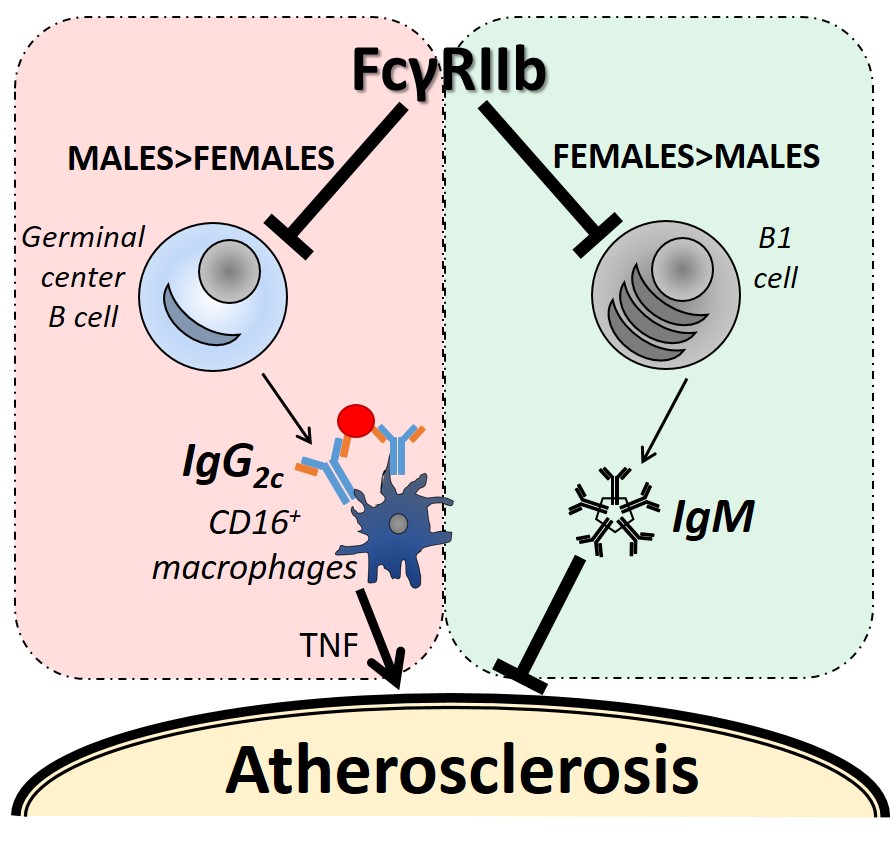

Supplement: Supplementary file 2 [file atv-39-1379-s002.jpg]
