# Supplementary material for: B Cell Fcγ Receptor IIb Modulates Atherosclerosis in Male and Female Mice by Controlling Adaptive Germinal Center and Innate B-1-Cell Responses
Source: Arterioscler Thromb Vasc Biol. 2019 May 16;39(7):1379–89. doi: 10.1161/ATVBAHA.118.312272 (PMC6636804; doi:10.1161/ATVBAHA.118.312272)
Supplement: Supplementary file 3 [file atv-39-1379-s003.pdf]

Chakraborty et al

B Cell FcγRIIb and Atherosclerosis

Supplemental Material

Supplemental Figure I

Pre-gated on FSc/SSc
and Live/Dead staining

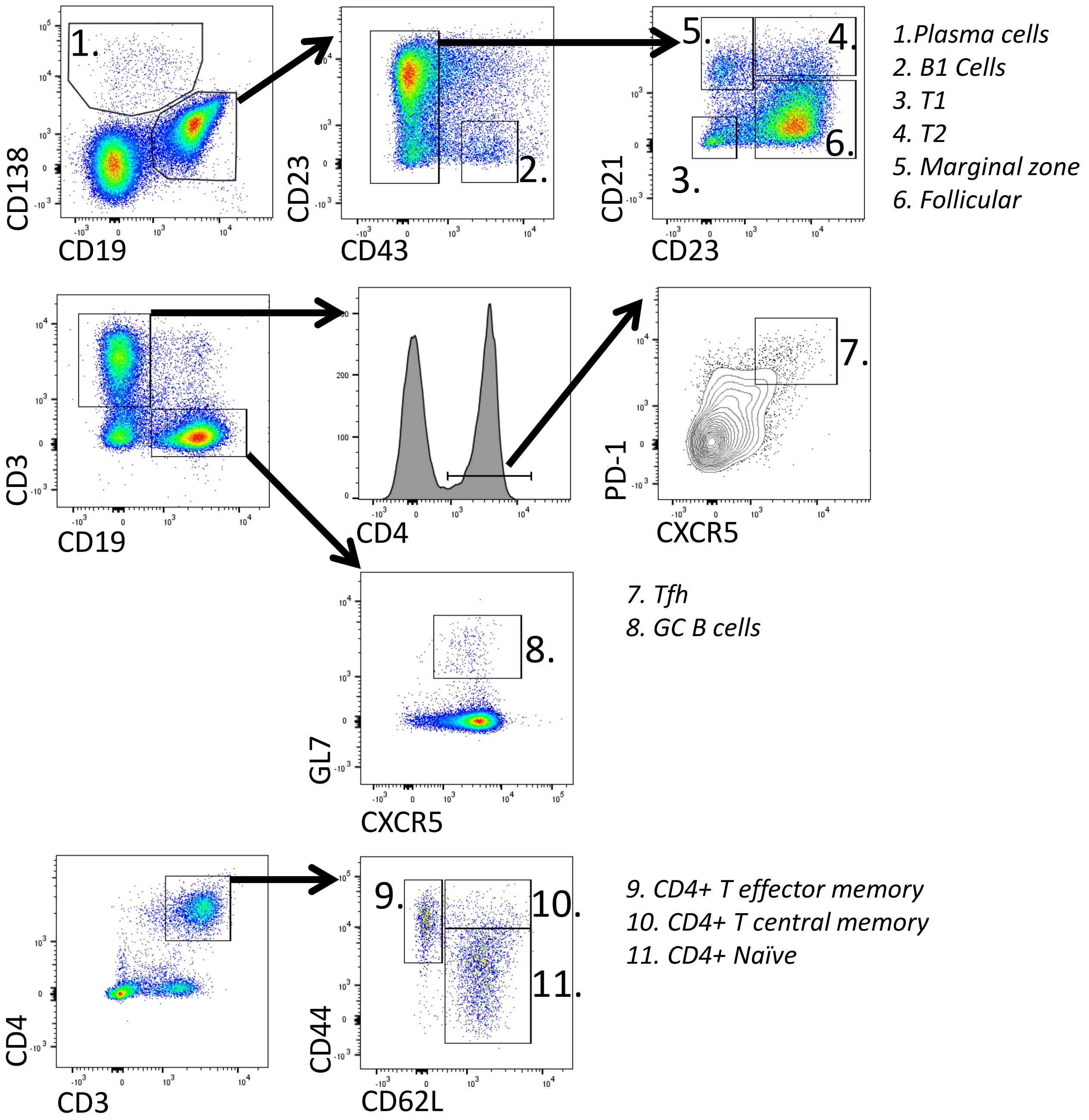

Supplemental Figure I. Gating strategies used in flow cytometry. In some experiments, GC B cells were gated as $B220^{+}$ IgM^{+} $CD95^{+}$ $GL7^{+}$. B1 cells are additionally $CD19^{+}$ IgM^{+} $B220^{lo}$.

Suplemental Figure II

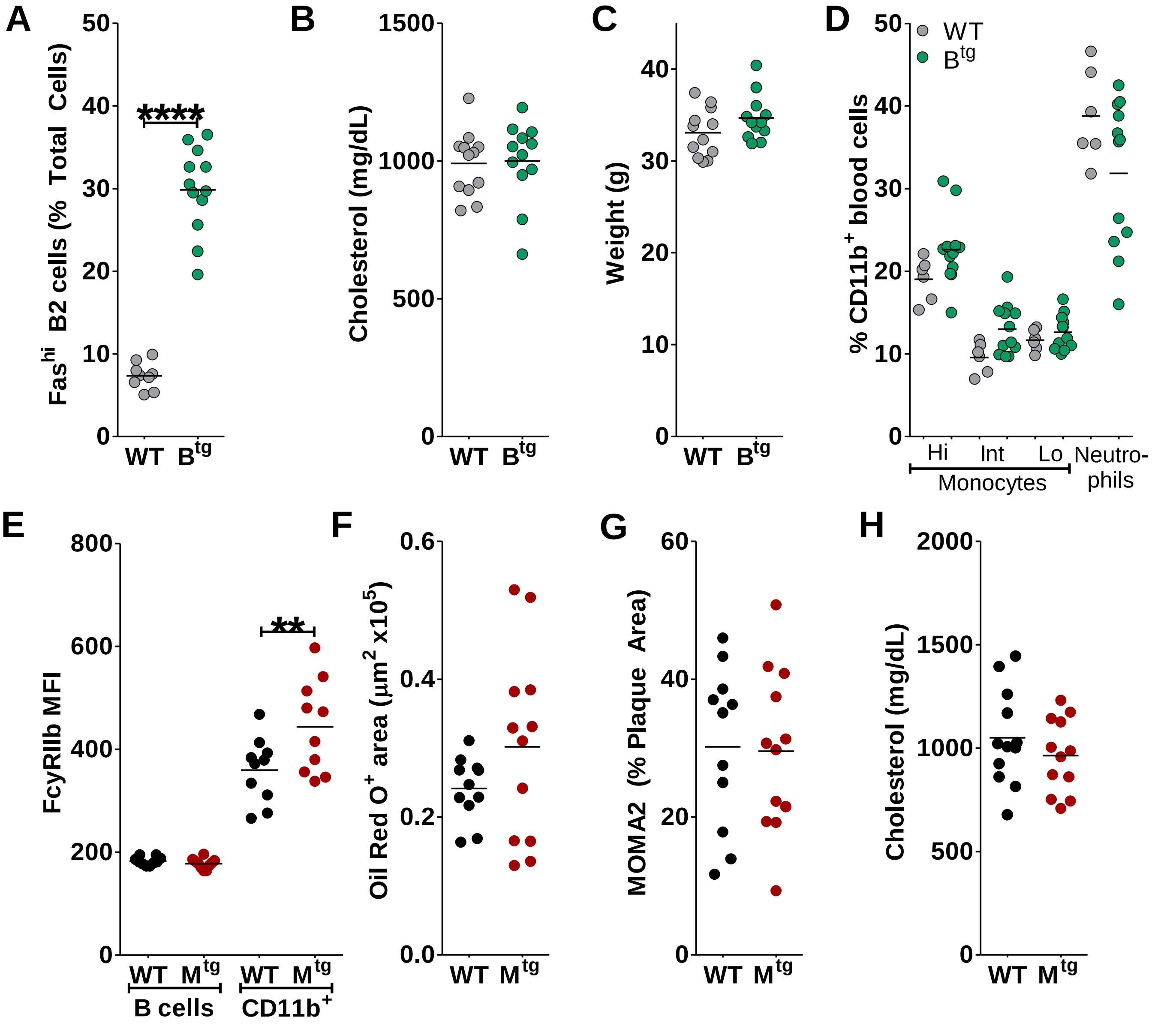

Supplemental Figure II. **A-D.** Analysis of *Ldlr*$^{-/-}$ chimeric mice transplanted with WT control or B^{tg} bone marrow after 6 weeks western diet. **A.** Proportion of B cells expressing high levels of Fas (CD95^{hi} GL7^{-}). **B**. Serum total cholesterol. **C**. Body weight. **D**. Blood monocyte subsets (CD11b^{+} Ly6G^{-} CD115^{+} and Ly6C lo, int or hi) and neutrophils (CD11b^{+} Ly6G^{+}). **E-H**. Analysis of *Ldlr*$^{-/-}$ chimeric mice transplanted with WT control or FcγRIIb-macrophage transgenic (M^{tg}) bone marrow after 6 weeks western diet. **E**. Mean flurorescent intensity for FcγRIIb on B cells and CD11b^{+} cells. **F**. Atherosclerotic plaque area. **G**. Macrophage (MOMA2)-positive area of plaque. **H**. Serum total cholesterol.

Supplemental Figure III

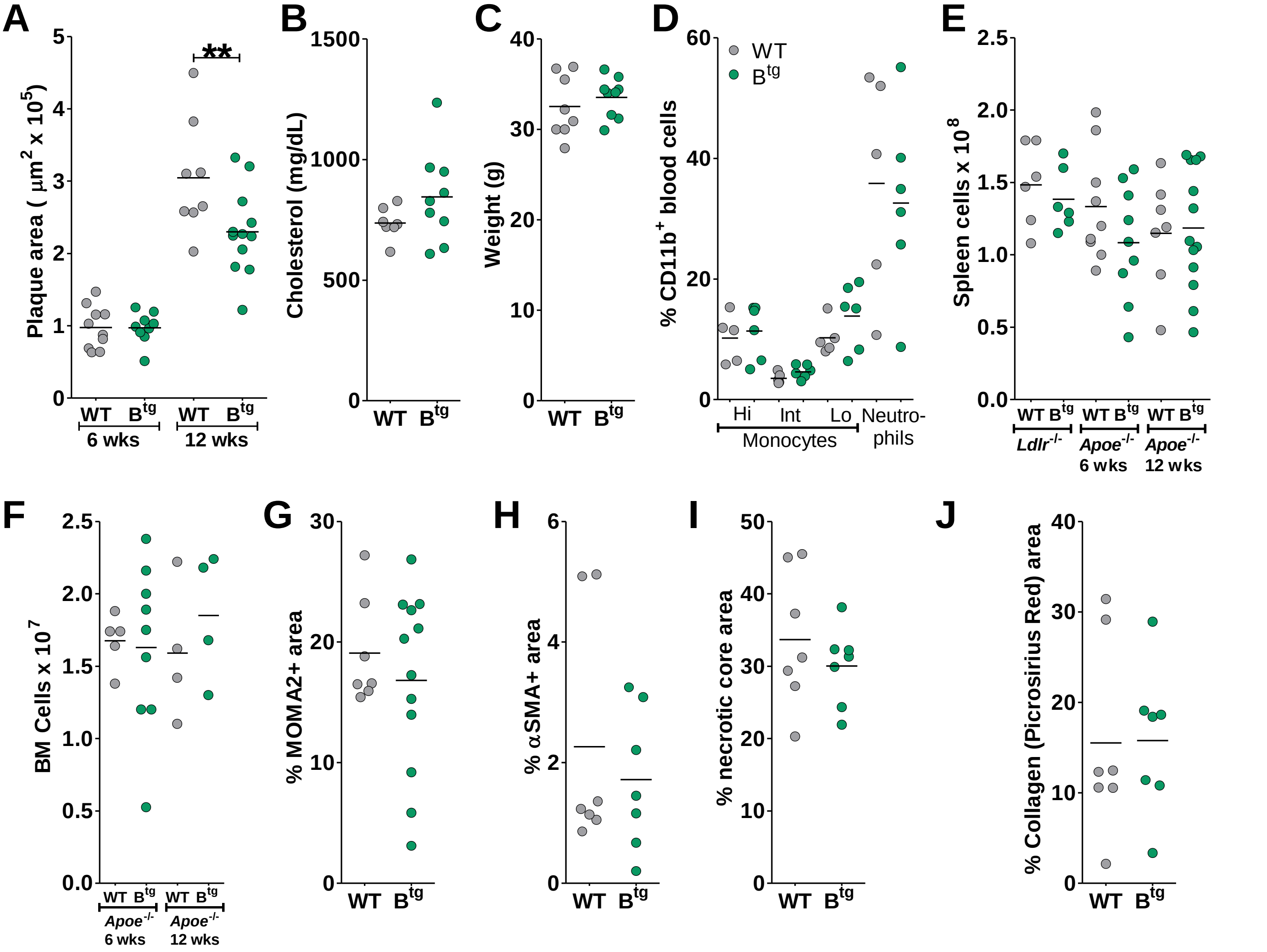

Supplemental Figure III. **A-D**. Analysis of *Apoe*$^{-/-}$ or B^{tg} *Apoe*$^{-/-}$ mice after 6 or 12 weeks western diet. **A**. Atherosclerosis quantified using Oil Red O-stained aortic root cryosections. **B**. Serum total cholesterol. **C**. Body weight. **D**. Blood monocyte subsets (CD11b^{+} Ly6G^{-} CD115^{+} and Ly6C lo, int or hi) and neutrophils (CD11b^{+} Ly6G^{+}). **E.** Total cell numbers in spleens from each B^{tg} cohort. **F**. Bone marrow cell numbers in *Apoe*$^{-/-}$ or B^{tg} *Apoe*$^{-/-}$ mice after 6 or 12 weeks western diet. **G-J**. Analysis of atherosclerotic plaques for proportions of positive staining for MOMA2 (G), α-SMA (H), necrotic core (I) or collagen (J) in aortic roots from *Apoe*$^{-/-}$ or B^{tg} *Apoe*$^{-/-}$ mice after 12 weeks western diet.

Supplemental Figure IV

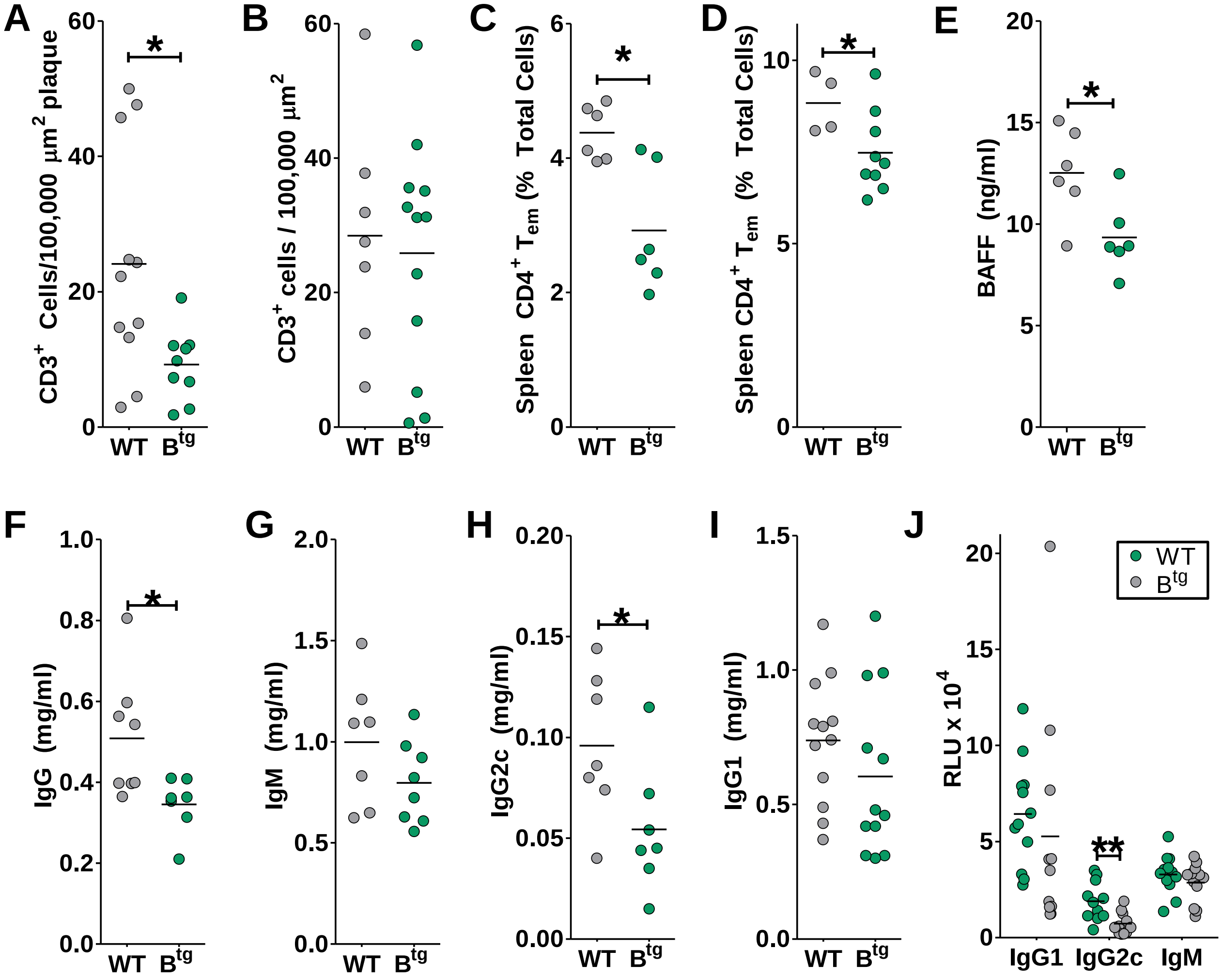

Supplemental Figure IV. **A+B**. Levels of plaque CD3+ T cells in aortic roots from *Ldlr-/-* chimeric mice transplanted with WT control or Btg bone marrow after 6 weeks western diet (A) *or Apoe-/-* or Btg *Apoe-/-* mice after 12 weeks western diet (B). **C-D**. Proportion of spleen effector memory CD4+ T cells (CD3+ CD4+ CD62L- CD44hi) in *Ldlr-/-* chimeric mice transplanted with WT control or Btg bone marrow after 6 weeks western diet (C) *or Apoe-/-* or Btg *Apoe-/-* mice after 12 weeks western diet (D). **E**. Serum BAFF levels in *Ldlr-/-* chimeric mice transplanted with WT control or Btg bone marrow after 6 weeks western diet. **F-J**. Serum antibody titres in *Ldlr-/-* chimeric mice transplanted with WT control or Btg bone marrow after 6 weeks western diet.

Supplemental Figure V

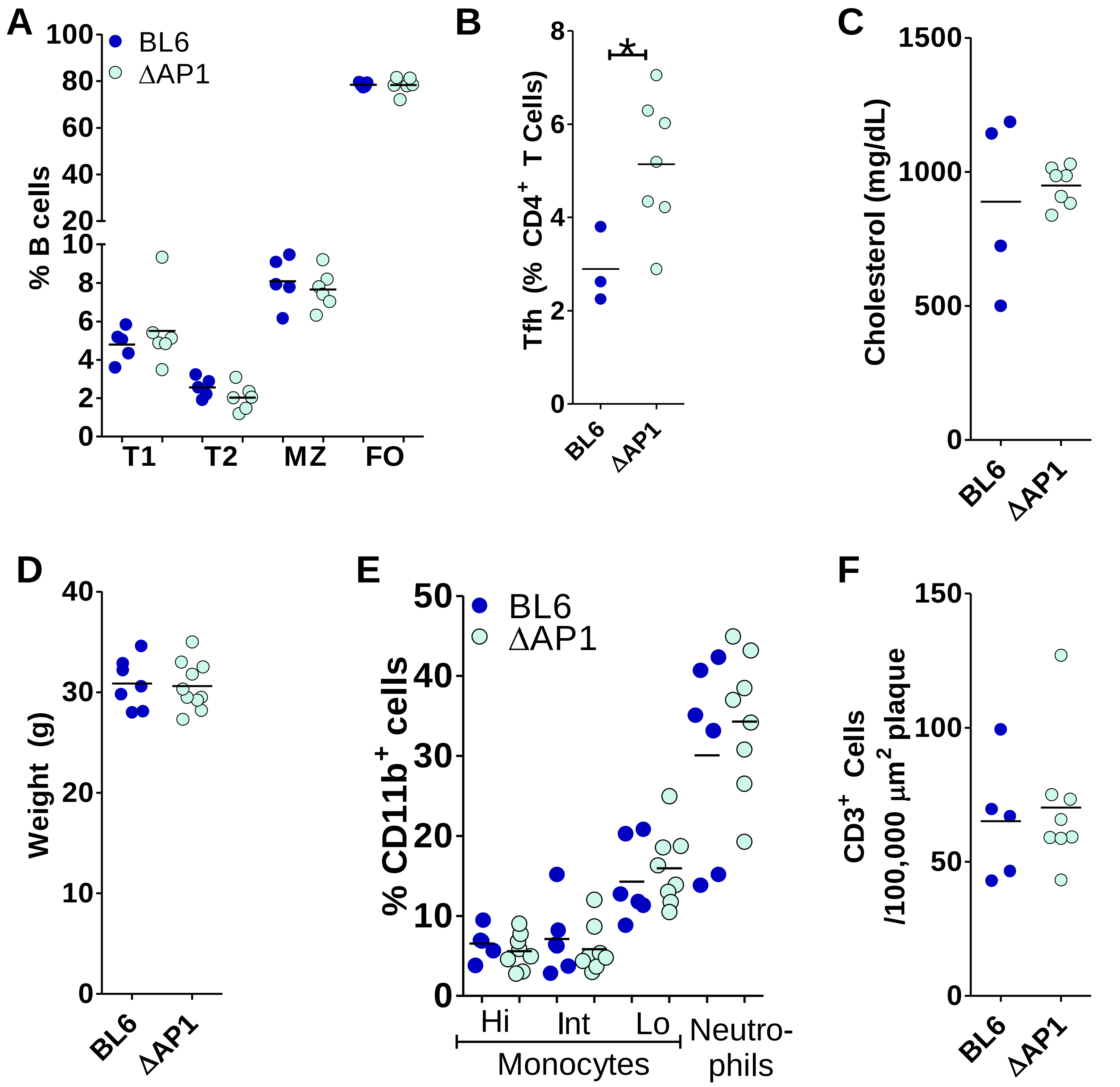

Supplemental Figure V. A-F. Analysis of male *FcγRIIb*^{BL6} *Apoe*$^{-/-}$ (BL6) or *FcγRIIb*$^{ΔAP-1}$ Apoe$^{-/-}$ (ΔAP-1) mice after 6 weeks western diet. Proportions of spleen B cell subsets (see figure S1). **B**. Follicular helper T cells (CD3$^+$ CD4$^+$ PD-1^{hi} CXCR5^{hi}). **C**. Body weights. **D**. Serum total cholesterol. **E**. Blood monocytes subsets (CD11b$^+$ Ly6G$^-$ CD115$^+$ and Ly6C lo, int or hi) and neutrophils (CD11b$^+$ Ly6G$^+$). **F.** Plaque CD3+ Cells. *p<0.05.

Supplemental Figure VI

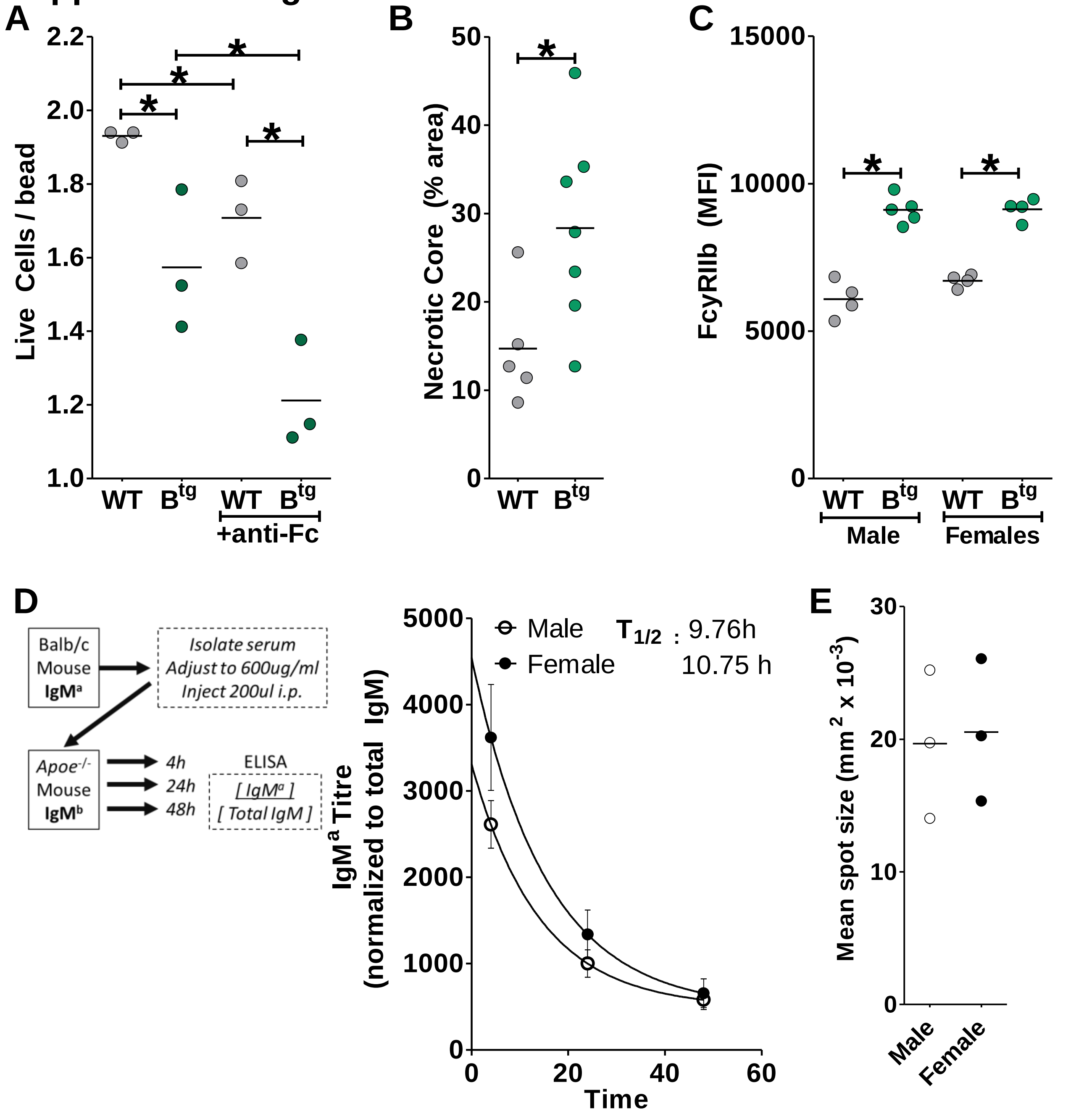

Supplemental Figure VI. A. B1 cells purified from peritoneum of *Apoe*$^{-/-}$ or B^{tg} *Apoe*$^{-/-}$ mice were cultured for 16h with or without anti-FcγRIIB (2.4G2 antibody) then stained with annexin V and propidium iodide. Absolute numbers of remaining live cells (AnnV^{-} PI^{-}) was quantified by flow cytometry using beads for normalization. **B**. Necrotic core area in aortic root plaques from female *Apoe*$^{-/-}$ or B^{tg} *Apoe*$^{-/-}$ mice after 6 weeks western diet. **C.** FcγRIIb levels on Spleen B1 cells in male and female *Apoe*$^{-/-}$ or B^{tg} *Apoe*$^{-/-}$ mice. **D**. IgM clearance rates determined after transfer of IgM^{a} serum (from Balb/c mice) into male or female *Apoe*$^{-/-}$ mice (each gender received gender matched serum). **E.** Mean spot size of IgM-secreting spleen cells from male and female *Apoe*$^{-/-}$ mice analysed by Elispot. Representative experiment of more than 5.

Supplemental Figure VII

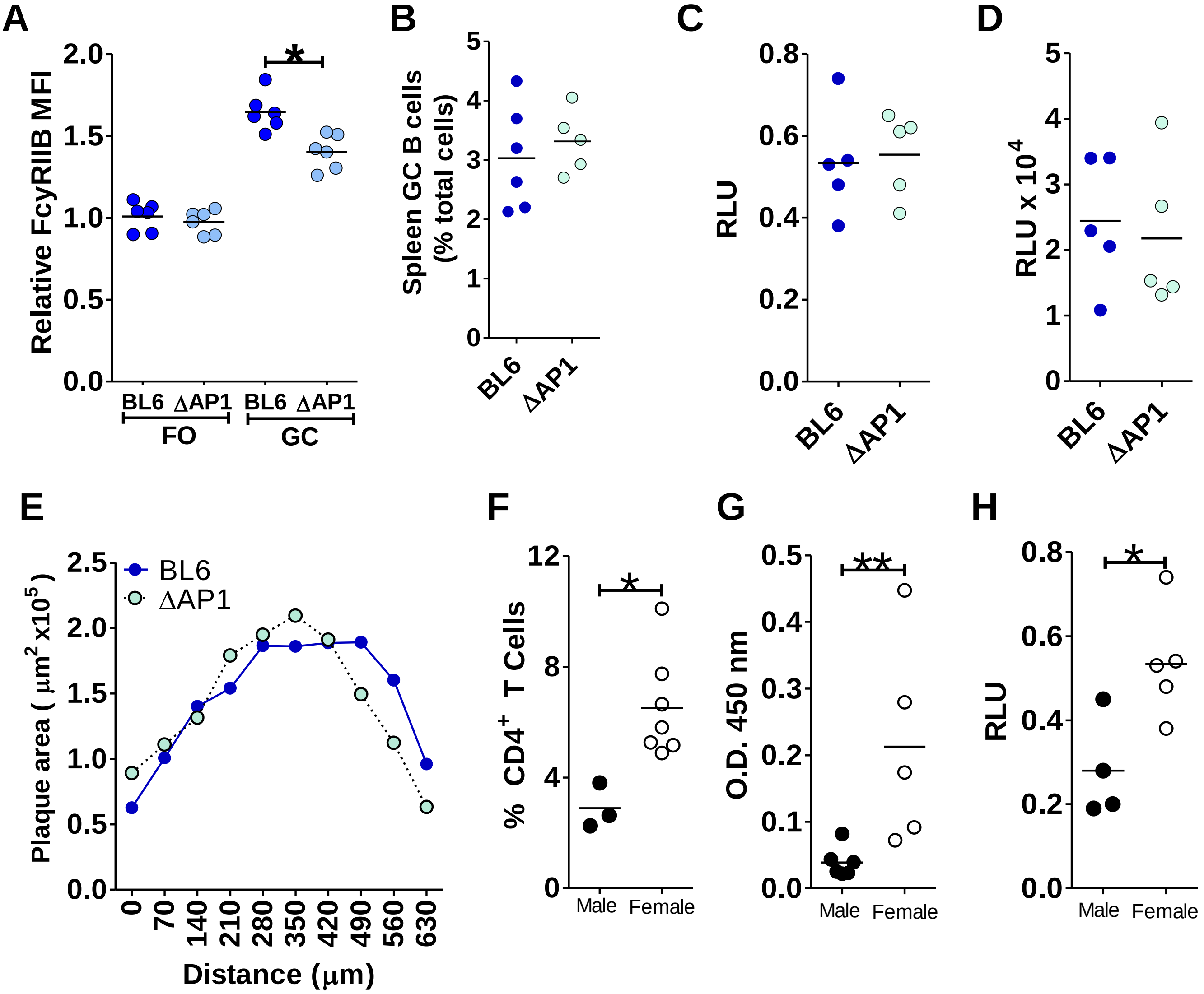

Supplemental Figure VII. A-E. Analysis of *Apoe*$^{-/-}$ or *FcγRIIb*$^{\Delta AP\text{-}1}$ *Apoe*$^{-/-}$ female mice after 6 weeks western diet. **A**. Mean fluorescence intensity (MFI) of anti-FcγRIIb staining on follicular (FO) or germinal center (GC) B cells from *Apoe*$^{-/-}$ or *FcγRIIb*$^{\Delta AP\text{-}1}$ *Apoe*$^{-/-}$ female mice. **B**. Spleen GC B cell levels. **C.** Total serum IgG2c. **D**. Total IgM levels. **E**. Atherosclerotic plaque area. N=6/group. *p<0.05. F-H. Comparison of male and female *Apoe*$^{-/-}$ mice. **F**. Follicular helper T cells. **G**. Anti-dsDNA IgG antiboides. **H**. Total IgG2c.

Supplemental Table I

Target	Clone	Company
**Ly6C**	7/4	AbD Serotec
**Ly-6G**	1A8	BD
**CD95 (Fas)**	Jo2	BD
**CD11b**	M1/70	Biolegend
**CD11c**	N418	Biolegend
**CD16.2**	9E9	Biolegend
**CD43**	Ly48 gp115	Biolegend
**CD44**	1M7	Biolegend
**CD62L**	MEL-14	Biolegend
**CD138**	281-2	Biolegend
**CD3**	145-2C11	Biolegend
**CD19**	6D5	Biolegend
**CXCR5**	L138D7	Biolegend
**PD-1**	4B12	Biolegend
**CD23**	B3B4	Biolegend
**CD16/32**	93	Biolegend
**MHCII**	M5/114.15.2	Biolegend
**B220**	RA3-6B2	Life Tech.
**CD4**	RM4-5	Life Tech.
**IgM**	II/41	Life Tech.
**GL7**	GL7	Life Tech.
**CD21**	4E3	Life Tech.
**CD115**	AFS98	Life Tech.
**Ki67**	SolA15	Life Tech.
**CD64**	REA286	Miltenyi

Supplemental Table II - Summary of effects of B cell FcγRIIB models on major parameters

FcγRIIb mutation	**FcγRIIB**^{Btg} *(increased FcγRIIB on B cells)*			**FcγRIIB**$^{\Delta AP\text{-}1}$ *(decreased FcγRIIb on GC B Cells)*	
Model	***Ldlr***$^{-/-}$	***Apoe***$^{-/-}$		***Apoe***$^{-/-}$	
Gender	**Male**	**Male**	**Female**	**Male**	**Female**
**Parameter:**					
Plasma Cells	n.d.	↓	↓*	↔	↔*
GC B Cells	n.d.	↓	↓*	↑	↔
B1 Cells	↔*	↔	↓	↔	↔*
IgG2c	↓	↓	↓*	↑	↔
IgG1	↓	↓	↓*	↔	↔*
IgM	↔	↔	↓	↑	↔*
Teff	↓	↓	↓*	↔	↔*
Atherosclerosis	↓	↓	↑	↑	↔
**Data not shown.*					
